# Adherence to Ketogenic and Mediterranean Study Diets in a Crossover Trial: The Keto–Med Randomized Trial

**DOI:** 10.3390/nu13030967

**Published:** 2021-03-17

**Authors:** Matthew J. Landry, Anthony Crimarco, Dalia Perelman, Lindsay R. Durand, Christina Petlura, Lucia Aronica, Jennifer L. Robinson, Sun H. Kim, Christopher D. Gardner

**Affiliations:** 1 Stanford Prevention Research Center, Department of Medicine, School of Medicine, Stanford University, Stanford, CA 94305, USA; matthewlandry@stanford.edu (M.J.L.); crimarco@stanford.edu (A.C.); daliap@stanford.edu (D.P.); lrdurand@stanford.edu (L.R.D.); cpetlura@stanford.edu (C.P.); laronica@stanford.edu (L.A.); jlrobinson@stanford.edu (J.L.R.); 2 Division of Endocrinology, Gerontology and Metabolism, Department of Medicine, Stanford University Medical Center, Stanford, CA 94305, USA; sunhkim@stanford.edu

**Keywords:** diet adherence, ketogenic, Mediterranean, dietary trial, crossover trial

## Abstract

Adherence is a critical factor to consider when interpreting study results from randomized clinical trials (RCTs) comparing one diet to another, but it is frequently not reported by researchers. The purpose of this secondary analysis of the Keto–Med randomized trial was to provide a detailed examination and comparison of the adherence to the two study diets (Well Formulated Ketogenic Diet (WFKD) and Mediterranean Plus (Med-Plus)) under the two conditions: all food being provided (delivered) and all food being obtained by individual participants (self-provided). Diet was assessed at six time points including baseline (×1), week 4 of each phase when participants were receiving food deliveries (×2), week 12 of each phase when participants were preparing and providing food on their own (×2), and 12 weeks after participants completed both diet phases and were free to choose their own diet pattern (×1). The adherence scores for WFKD and Med-Plus were developed specifically for this study. Average adherence to the two diet patterns was very similar during both on-study time points of the intervention. Throughout the study, a wide range of adherence was observed among participants—for both diet types and during both the delivery phase and self-provided phase. Insight from this assessment of adherence may aid other researchers when answering the important question of how to improve behavioral adherence during dietary trials. This study is registered at clinicaltrials.gov NCT03810378.

## 1. Introduction

The prevalence of type 2 diabetes mellitus (T2DM) is increasing at an alarming rate in the United States. According to latest national estimates, just over one in ten US adults have T2DM and one in three adults have prediabetes [1]. Strong evidence supports dietary modification as an efficacious and cost-effective strategy to prevent and manage T2DM [2,3]. The types and amounts of dietary carbohydrates within a recommended dietary pattern have been established as important factors in both prevention and treatment [4]. However, the optimal amounts and sources of carbohydrates remain unclear. 

The average carbohydrate intake in the US is in the range of 45–50% of daily energy, with low-carbohydrate (low-carb) diets providing less than 40% of daily energy intake from carbohydrates [5,6]. There are many approaches to a low-carb diet. A Mediterranean diet promotes the consumption of high fat sources such as olive oil, nuts, seeds, avocados and fatty fish, and as a consequence is a moderately low-carb diet pattern, in the range of 35–40% energy from carbohydrates [7,8]. The carbohydrate sources promoted by a Mediterranean diet are of high nutritional quality and include legumes, fruits, whole grains, and nonstarchy vegetables. In comparison, a Ketogenic diet is the most restrictive low-carb diet, in the range of 5–10% energy from carbohydrates [9]. That level of carbohydrate restriction requires eliminating legumes, fruits, starchy vegetables, and grains (including whole grains) from the diet. Both the Mediterranean and Ketogenic diets have been found to improve glycemic control and other diabetes-related outcomes [10,11,12,13,14]. An important practical question is whether the diets differ in long-term sustainability. Long-term adherence to diet patterns is important in maintaining any health changes achieved through dietary changes [15,16], and there is a paucity of data on comparative adherence to low-carb diets that differ in their level of carbohydrate restriction.

Adherence (i.e., the extent to which participants meet study goals and follow defined and recommended study diet patterns) is an important factor to consider when interpreting study results from randomized controlled trials (RCTs) comparing one diet to another. However, adherence is often inadequately reported [17]. The literature on adherence in dietary interventions is limited, and the assessments that have been published tend to focus on metrics, such as attendance to instructional sessions, rather than actual fidelity to the food and nutrient composition of the study diets. It has been suggested that dietary adherence may be more important than the macronutrient composition of diets for the long-term improvement and maintenance of disease-related outcomes (i.e., improved glycosylated hemoglobin (HbA1c) or weight) [15,18,19,20]. 

The primary objective of this Ketogenic vs. Mediterranean (Keto–Med) randomized, crossover trial was to contrast HbA1c levels among adults with either T2DM or prediabetes after 12 weeks of assignment to a Ketogenic diet, and 12 weeks of assignment to a Mediterranean diet. All food was intended to be provided for the first four of the 12 weeks of each phase (some COVID-19-related disruptions occurred), with food purchase and preparation being the responsibility of participants during the latter eight weeks of each 12-week phase. Extensive diet assessment data were collected, including a final set of data collection 12 weeks after participants completed both intervention phases, during which time participants received no advice or contact from study staff. The purpose of this secondary analysis was to provide a detailed examination and comparison of adherence to the two study diets under the two conditions of food being provided (delivered) or food obtained by individual participants (self-provided), as well as an assessment of which of the two diet patterns more closely resembled what participants were consuming 12 weeks after the intervention portion of the study ended.

## 2. Materials and Methods

### 2.1. Description of the Keto–Med Trial

The purpose of the Keto–Med randomized, crossover trial was to compare two metabolically distinct diets, the Well Formulated Ketogenic Diet (WFKD) vs. Mediterranean-Plus Diet (Med-Plus), defined in detail below, among individuals with T2DM and prediabetes, for their effects on metabolic health. The primary objective was to determine which diet is more effective for improving blood glucose control, and to explore whether diabetes status (T2DM vs. prediabetes) was an effect modifier. Those outcomes are the focus of a separate publication (in process). The trial was designed as a pilot study, and sample size was determined by resources. This analysis focuses on adherence and satisfaction to the two dietary phases of the Keto–Med protocol. Participant enrollment began on 5 June 2019 and continued through 21 February 2020. The date of final follow-up data collection was 4 December 2020. The study design is illustrated in Supplemental Figure S1. 

### 2.2. Modifications to the Study Design Due to COVID-19

A portion of this study was impacted by the shelter-in-place orders (initiated 16 March 2020) that resulted from the COVID-19 pandemic. A full description of modifications to the study made by the investigators and study staff are described in Supplemental Methods File S1. Briefly, participants whose end-of-phase blood draws fell during the initial shelter-in-place order (mid-March to end of May) were asked to extend the duration of their assigned diets until research staff were able to restart in-person blood draw clinic visits in early June. Data collection involving online surveys, phone, or mail continued during the shelter-in-place—i.e., participants were provided with additional continuous glucose monitors (CGMs) & stool kits through the mail, and extra dietary recalls were collected.

### 2.3. Participants

The target population was generally healthy adults ≥18 years of age with a diagnosis of T2DM (HbA1c ≥ 6.5% or fasting glucose of ≥126 mg/dL) or prediabetes (HbA1c ≥ 5.7% or fasting glucose of ≥100 mg/dL) [21]. Participants were recruited from the San Francisco Bay area. Recruitment strategies used by research staff included mass mailings to existing study participant lists and patient referrals from the clinic of coinvestigator, Sun Kim, MD. Participants were required to have a stable dietary history, defined as neither including nor eliminating a major food group within their diet for at least the previous month.

Exclusion criteria were weighing <110 lbs (45 kg); having a BMI (kg/m^2^) ≥ 40; LDL cholesterol > 190 mg/dL; systolic blood pressure > 160 mmHg or diastolic blood pressure > 90 mmHg. A full description of exclusion criteria is available at clinicaltrials.gov (NCT03810378). 

### 2.4. Randomization

Once participants completed both the online and in-person screeners to determine initial eligibility, consented to the study, and completed baseline measurements to determine final eligibility and diabetes status, they were randomly assigned to a diet sequence. One group was assigned to WFKD in Phase 1 and Med-Plus in Phase 2. The other group was assigned the opposite order. The randomization process was stratified by T2DM vs. prediabetes status. Diet randomization was performed using an allocation sequence determined by computerized random-number generation in block sizes of 4 by a statistician not involved in intervention delivery or data collection. Participants did not learn of their diet group assignment until they completed all baseline measures. 

### 2.5. Dietary Intervention

The intervention component of the study involved having all participants change their eating habits twice, trying to achieve and maintain two dietary patterns, WFKD and Med-Plus, for 12 weeks each. A primary dietary objective of the intervention was to guide study participants to be adherent to two sets of dietary guidelines that shared three important similarities and differed in three important characteristics (Table 1). 

During the WFKD phase, participants were counseled to sustain nutritional ketosis by limiting carbohydrates to 20–50 g/day, keeping fats as close to 70% of daily calories as possible, and keeping proteins to no more than 1.5 g/kg ideal body weight/day. This criteria is based on the recommendations of Volek and Phinney [22], with the additional dietary instruction of including consumption of >3 servings/day of nonstarchy vegetables and adequate mineral and fluid intake for the ketogenic state. Whole foods were promoted, mineral supplements were not encouraged, and all processed foods were strongly discouraged. 

During the Med-Plus phase, participants were encouraged to sustain a Mediterranean diet based on recommendations from the Mediterranean Diet Pyramid [23], with the additional restriction of no added sugars or refined grains. Instructions were to follow a mostly plant-based diet that included vegetables (including starchy vegetables), intact whole grains, whole fruits, legumes, nuts, and seeds, with fish being the primary animal protein, and olive oil the primary fat. As in the WFKD, whole foods were promoted, and all processed foods were strongly discouraged. There was no prescribed washout period between intervention phases. 

Participants were provided with diet education and with reference amounts of diet components that they should consume per day or per week (Supplemental Methods Files S2–S5). The instructions did not include a prescribed caloric deficit; rather participants were told to eat ad libitum on both diets. Participants received weekly individual nutrition counseling and education sessions conducted by a health educator (registered dietitian and certified diabetes educator). These sessions were conducted via email or phone with a face-to-face meeting every 4 weeks, during transition times. This in-person meeting changed to videotelephony (Zoom Video Communications, San Jose, CA, USA) after the start of the COVID-19 pandemic. Throughout each diet phase, educators monitored logged food intake (logged by participants via Cronometer, (Cronometer Pro, Nutrition Tracking Software for Professionals; https://cronometer.com/pro) and blood ketone status (when on WFKD). Participants needing extra support or expressing greater interest in learning more about their assigned diet were provided with additional sessions. 

### 2.6. Collection of Dietary Intake

Two types of dietary data were collected. For the primary reporting data, three unannounced 24-h dietary recalls (24 h DR) were administered within a one week window (2 weekdays and one weekend day) of each major time point via telephone by a trained Registered Dietitian using Nutrition Data Systems for Research (NDS-R) (Nutrition Coordinating Center, Minneapolis, MN, USA, 2019), a computer-based software application that facilitates the collection of recalls in a standardized fashion [24]. The NDS-R database includes over 18,000 foods, including 7500 brand name products; ingredient choices and preparation methods provide 160,000+ food variants. Additionally, NDS-R can generate values for 178 nutrients, nutrient ratios and other food components, and food group assignments are also provided. Dietary intake data gathered by interview was governed by a multiple-pass interview approach. Five distinct passes provided multiple opportunities for the participant to recall food intake. A “Foods Amounts Booklet” was used to assist participants with portion size estimation. Recalls conducted took between 15–45 min to complete, depending upon the complexity of the intake. Diet assessors had a wide range of availability to accommodate participants’ varying schedules, from early morning through evening. Generous staff time was required for capturing participant recalls. Quality assurance was performed on all dietary recall data by research staff trained in NDS-R. 

Secondly, throughout the study, participants were encouraged to log their food intake using Cronometer. Participants were instructed to use the app for three purposes: (1) to increase their awareness of their daily dietary intake; (2) as a communication tool with the health educators and; (3) to have a log of foods consumed to later be correlated with blood glucose (from CGM) in response to the meals. Educators reviewed entries and made dietary suggestions based on current reported intake. 

### 2.7. Food Delivery

Participants were provided with all meals and snacks for the first 4 weeks of each phase of the study at no cost to them (i.e., weeks 1–4 and weeks 13–16) (see Supplement Methods File S1 for modifications due to COVID-19). Meals were provided by a San Francisco Bay Area food delivery service (Methodology; https://www.gomethodology.com/). Research staff worked closely with the delivery company to develop a set of menu offerings that exemplify a high-quality ketogenic diet and a high-quality Mediterranean diet. Meals were delivered once per week, with 7 days’ worth of menu items per delivery, for the first four weeks during each diet phase. A list of foods provided by Methodology for both diets are provided in Supplemental Table S1. 

Rather than tailoring the weekly deliveries to individually determined energy intake levels, all participants received a set of menu items designed to provide ~2800 kcal/day for seven days. This level was intended to be adequate to provide even the largest and most active participant in the study with adequate energy intake to be sustained without hunger. Importantly, it was expected and explained that most participants would not eat all of the food. They were instructed to consume an equal proportion of each menu item over the course of the week to maintain adequate balance of nutrients. For example, if they were consuming 50% of the fish menu item over the course of the week, they were instructed to consume 50% of each of the other dishes over the course of the week. Each recipe was provided in a separate jar and was prepopulated into the Cronometer application, so participants were able to log the proportion of the jar they consumed (e.g., ½ jar or ¾ of the jar). The health educators reviewed these entries and advised participants if proportions of items consumed needed adjustments. 

For the WFKD phase, recommendations from Volek and Phinney [22] were followed and adapted based on the food delivery company’s delivering capabilities. One notable characteristic of the food delivery service was that it was a dairy-free (with the exception of ghee) and gluten-free operation. The final WFKD menu offerings provided participants a diet with: 66% fat, 5% carbohydrate, and 29% protein. For the Med-Plus phase, recommendations from the Mediterranean Pyramid [23] were followed and included the extra restriction of no refined grains and no added sugars. The final menu offerings provided participants a diet with 46% fat, 38% carbohydrate, and 16% protein. 

The study was designed with an initial 4 weeks of food delivery to allow for immediate, high level adherence at the start of each study phase. In addition, it was expected that after 4 weeks of food delivery and health educator counseling, the study participants would have a solid understanding of the amounts and types of foods they should purchase and prepare on their own in order to achieve maximum adherence to the study diets. 

### 2.8. Measure of Adherence to Well Formulated Ketogenic Diet (WFKD) and Mediterranean-Plus Diet (Med-Plus)

An average of all available dietary recalls and records at each time point was used to calculate adherence scores. Adherence scores were calculated at six time points from NDS-R diet data to show degree of adherence to the recommended dietary recommendations. The six time points included baseline (x1), week 4 of each phase when participants were still receiving food deliveries (x2), week 12 of each phase when participants were providing and preparing food on their own (x2), and 12 weeks after participants completed both diet phases and were free to choose their own diet pattern (x1). 

The WFKD adherence score consists of 5 components: total carbohydrates, percent of calories from fat, nonstarchy vegetables, added sugars, and refined grains. Low-carb intake is the key tenet of a ketogenic diet; therefore, net carbohydrate intake was weighted to make up half of the final score. Participants were given between 0–4 points based on how well their average intake matched recommended carbohydrate intake. A total possible score was derived by multiplying the points for each component by its scoring factor then summing the 5 component scores so that the maximum composite score was 10. A higher score reflects better adherence. The scoring cutoffs for each of the components are provided in Supplemental Table S2. 

The Med-Plus adherence score consists of 8 components. Diet components that were most heavily emphasized by health educators during instruction were: inclusion of nonstarchy vegetables, intact whole grains and starchy vegetables, and limiting added sugars and refined grains. The contribution of these 4 components to the adherence score were more heavily weighted than the other components. The remaining four components, including fruits, legumes, fish, and restricting red meat (beef, pork, and lamb), were given a smaller weighting. Participants were given between 0–4 points based on how well their average intake matched recommended intakes for each of the 8 primary components. A total possible score was derived by multiplying the points for each component by its scoring factor then summing the 8 component scores so that the maximum composite score was 10. A higher score reflects better adherence. The scoring cutoffs for each of the components are provided in Supplemental Table S3. 

The weekly meals, as designed and provided by the food delivery service for both the WFKD and Med-Plus diet, was assigned full points (10 out of a possible 10) on both adherence scoring scales. 

### 2.9. Measurement of Ketone Levels

While participants were completing the WFKD phase of the study, they were provided with blood ketone monitors and strips (Abbott Precision Xtra Blood Glucose & Ketone Monitoring System) to measure ketones three times/week before breakfast (fasting state). Health educators reviewed these values with participants and used these to adjust carbohydrate intake recommendations to try to achieve a ketone range of 0.5–3.0 mmol/L. Ketone levels of 0.5 to 1.5 mmol/L indicate light nutritional ketosis and levels from 1.5 to 3.0 mmol/L indicate optimal nutritional ketosis [22]. Additionally, participants completed a fasting venous blood draw at seven time points throughout the study, prebaseline, baseline, 4 weeks, 12 weeks (end of diet 1), 16 weeks, 24 weeks (end of diet 2), and 36 weeks (follow-up). β-hydroxybutyrate was analyzed by the Core Laboratory for Clinical Studies Washington University School of Medicine (St. Louis, MO, USA) on EDTA plasma samples. Levels were measured by an enzymatic colorimetric method using β-Hydroxybutyrate Liquicolor reagents from Stanbio Laboratory (distributed by Sekisui Diagnostics) and were ran on a Roche cobas c501 analyzer.

### 2.10. Measurement of Satisfaction and Preparedness to Follow Study Diets

#### 2.10.1. Food Satisfaction

At the end of each diet phase, participants were asked to rate the overall level of service (e.g., delivery, packaging, labeling), overall taste, and overall quality of food products while receiving food deliveries. When participants were no longer receiving food deliveries and were purchasing and preparing foods on their own, they were asked to rate their general understanding of following the diet they were on, frequency of meals prepared at home versus meals eaten outside of the home, who was preparing meals, satisfaction with the study diet (taste and variety), and cost of foods for the diet phase compared to their prestudy diet. When they completed both diets, participants were asked to compare them in terms of taste and enjoyment, ease of following and affordability. Questionnaire items are provided in Supplemental File S6.

#### 2.10.2. Diet Satisfaction

Participants were also asked to rate their overall dietary satisfaction at 4 time points: baseline, end of diet 1, end of diet 2, and 12-week follow-up using the Diet Satisfaction Questionnaire (DSat-28©) [25]. Questions were grouped by categories, which included whether they felt they were living a healthy lifestyle, were eating outside the home more or less often, overall cost of the diet, preoccupation with food, and the planning and preparation needed for the diet. Questionnaire items are provided in Supplemental File S6.

#### 2.10.3. Qualitative Comments about Study Diets

At the conclusion of the study, participants were asked by health educators what aspects of each diet they preferred. 

### 2.11. Assessment of COVID-19 Related Alterations

Participants were asked to rate their ability to follow assigned diets during the pandemic shelter-in-place order, extent of stressors related to the pandemic, and changes in physical activity due to the pandemic. Questionnaire items are provided in Supplemental File S6.

### 2.12. Data Management

Study data were collected and managed using REDCap (Research Electronic Data Capture) [26,27]. The Stanford REDCap platform (http://redcap.stanford.edu) is developed and operated by Stanford Medicine Research IT team. REDCap is a secure, web-based software platform designed to support data capture for research studies, providing: (1) an intuitive interface for validated data capture; (2) audit trails for tracking data manipulation and export procedures; (3) automated export procedures for seamless data downloads to common statistical packages; and (4) procedures for data integration and interoperability with external sources.

### 2.13. Statistical Analysis

This secondary analysis was largely descriptive. Basic summary statistics (means, standard deviation and range) were used to describe baseline demographic characteristics, diet composition on both diets, and change in adherence to study diets. Matched-pairs *t*-tests were used to quantitatively compare adherence score means to both Keto–Med Diet Phases by timepoint. A *p*-value of 0.05 was used to denote significance. Change in adherence by study phase were depicted graphically using line graphs and butterfly charts. Data were analyzed and graphed using RStudio (Version 1.2.5042, RStudio Team, 2020). 

## 3. Results

### 3.1. Demographics

Table 2 presents baseline demographic characteristics. Of the 381 potential participants that completed an initial online screener for the study, 42 participants were randomly assigned to the intervention arms (Supplemental Figure S2). Two of the 42 randomized participants dropped out before beginning their first dietary phase. Four of the remaining 40 participants discontinued the study due to COVID-19 and one participant discontinued for unknown reasons. Data from participants who discontinued the study were used until time of dropout. The participants were approximately half female (55%), half Non-Hispanic white (48%), and mostly college educated (83%). Fifty-three percent of participants had prediabetes and 47% had T2DM. BMI ranged from 22.7–39.7 kg/m^2^; age ranged from 41–77 years.

### 3.2. Description of Food Delivery Meals

Macronutrient composition of foods during the food delivery phase as designed, as delivered, and as consumed by participants are provided in Table 3. The food delivery company was able to deliver foods that closely matched the macronutrient distribution designed by the research team. For foods consumed by participants, caloric intake was lower than expected on both diets, even though participants were not told to restrict intake. However, even with lower total energy, the percent macronutrient distribution of foods consumed were largely similar to as designed and as delivered distributions. 

### 3.3. Description of Diet Composition

**Figure 1:**
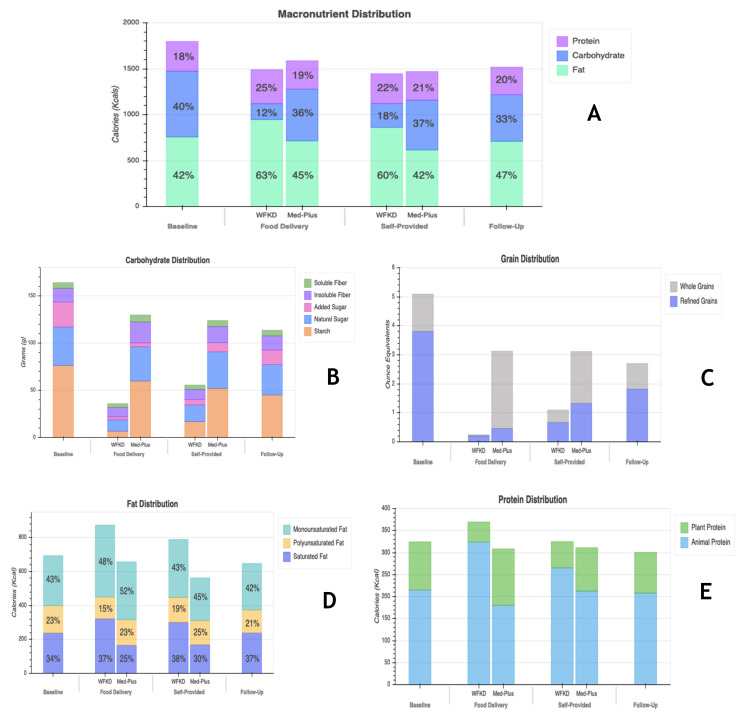
Nutrient data for means of (**A**) macronutrients, (**B**) carbohydrates, (**C**) whole and refined grains, (**D**) fats, and (**E**) animal and plant proteins at four time points (baseline, food delivery, self-provided and follow-up). Two bars are shown for food delivery and self-provided phases for comparison of data on each of the study diets (WFKD and Med-Plus).

Nutrient data for means of macronutrients, carbohydrates, whole and refined grains, fats, and animal and plant proteins, at major study time points, are shown in Figure 1A–E. Nutrient data at each study timepoint presented separately by randomization order are shown in Supplemental Figure S3A–E. Although there was no prescribed caloric restriction, participants reported lowering total calorie intake during food delivery and self-provided phases of both diets relative to baseline. The biggest difference in macronutrient composition of the diet was the carbohydrate:fat ratio. All phases, including baseline, were ≤40% in carbohydrates. Intake of carbohydrates dropped on both diets from baseline levels, but to a much greater extent during the WFKD phase. Added sugar as a part of total carbohydrate distribution decreased for both diets during the food delivery phase, was largely maintained at low levels through the self-provided food phase but increased at follow-up. Compared to baseline, consumption of whole and refined grains dropped during the WFKD phase when participants were receiving food deliveries. During the Med-Plus food delivery phase, participants reduced grain consumption to a lesser extent than did WFKD, but the reduction was primarily in refined grains; grains consumed during Med-Plus were primarily whole grains. During the self-provided phase of both diets there was some recidivism back to baseline levels with increases in grains coming largely from refined grains. While on the WFKD, participants consumed more fat than at baseline, and more fat than when on the Med-Plus diet at both the food delivery and self-provided phases. At 12-weeks follow-up, average grain consumption was still >2-ounce equivalent servings lower than baseline levels across all participants. During the food delivery phase, participants on the WFKD shifted to higher and more animal-based protein consumption compared to during the food delivery phase of the Med-Plus diet. Other than this, for all time points and both diets, the amount and proportions of protein were similar. 

### 3.4. Adherence to Study Diets

**Figure 2:**
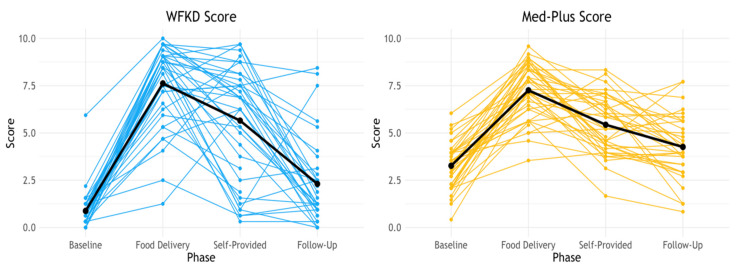
Individual participant change in diet adherence from baseline, food delivery, self-provided, and 12 weeks postintervention (follow-up). Solid black line represents the mean. While 23/36 (64%) participants received food deliveries during both diet phases, 13/36 (36%) participants only received food deliveries during their first diet phase due to disruptions in the study protocol caused by COVID-19.

Average and range of adherence scores for the two diet phases at the two intervention time points are provided in Table 4. Individual scores, including the baseline and follow-up time points are shown in Figure 2. The baseline scores for WFKD (0.9 ± 1.0; range 0.0–5.9) and Med-Plus (3.3 ± 1.2, range 0.4–6.0) were calculated and presented for the purpose of illustrating the magnitude of change participants underwent to shift from their prestudy diets to their intervention diets. The 12-week postintervention follow-up scores for WKFD (2.3 ± 2.3; range 0.0–8.4) and Med-Plus (4.3 ± 1.7; range 0.8–7.7) were calculated and presented similarly for the purpose of illustrating which of the two diet patterns the participants were closer to following after having mastered and experienced both of them during the intervention phase. The prestudy/baseline diets were not well aligned with either WFKD or Med-Plus, but of the two they were more similar to Med-Plus. The follow-up diets were not well aligned with either WFKD or Med-Plus, but as with baseline, more closely resembled the Med-Plus guidelines than the WFKD guidelines. Within each diet type, the adherence scores were statistically significantly different for all of the major time points compared to one another; i.e., baseline, food delivery, self-provided and follow-up, were all different from one another for WFKD scores and for Med-Plus scores (all *p*-values ≤ 0.003) Supplemental Table S4.

While receiving food deliveries, adherence to both study diets increased and was similar for both diet patterns. Adherence for both diets decreased by a similar amount for both diet patterns during the self-provided food phase, and, again, the scores were similar. At 12-weeks postintervention, there was greater recidivism to baseline diet patterns from the WFKD compared to Med-Plus. However, both the Med-Plus and the WFKD scores were higher at follow-up than at baseline. 

As illustrated in Figure 2, there was considerable variability in adherence scores among participants for both diet patterns while on-study and at follow-up. The variability was highest when participants were on the WFKD and were choosing foods on their own. While average adherence to either diet decreased from the food delivery to self-provided food phase, adherence to either diet actually increased for a select number of participants. 

**Figure 3:**
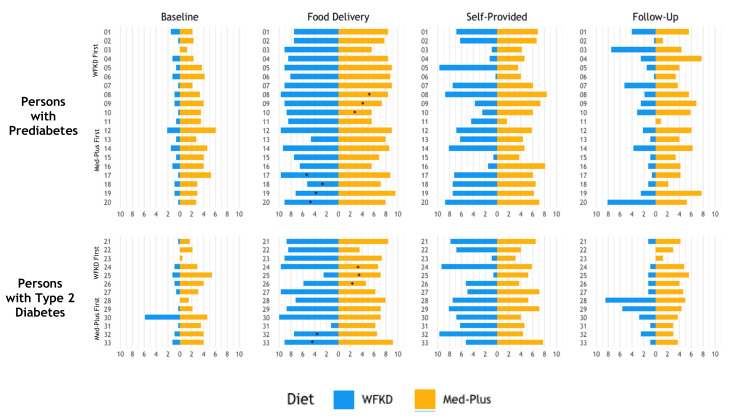
Comparisons of individual adherence during each phase of the study (baseline, food delivery, self-provided and Table 2 diabetes mellitus (T2DM). Data are shown for participants with data at all four time points. Due to disruptions in the study protocol caused by COVID-19, participants who did not receive food deliveries during their second diet phase are denoted with an asterisk (*).

Comparisons of individual adherence during phases of the study (baseline, food delivery, self-provided, and 12-week follow-up) for each diet (WFKD and Med-Plus) stratified by participants with prediabetes and T2DM are shown in Figure 3. Of participants with follow-up data, the majority (29/34, 85%) reported dietary intakes that were more similar to the Med-Plus than the WFKD diet at 12-weeks postintervention.

### 3.5. Ketone Blood Levels

Average weekly blood ketones of participants during the food delivery and self-provided phases of the WFKD ranged from 0.49 to 1.4 (Supplemental Figure S4). Participants were asked to measure and report their blood ketones to the research team via a mobile app. Some participants reported more frequently than others; on average participants had 22 (range 2–64) measurements. The number of measurements provided per week also ranged from 0-7. Fourteen percent of participants had at least one measurement during each week of the WFKD, 36% had at least one measurement for at least 10 of the weeks, 33% had at least one measurement for at least 7 of the weeks, and 17% had at least one measurement for fewer than 7 of the weeks. For 85% of weeks, the mean blood ketones for participants were within the light nutritional ketosis range (0.5–1.5 mmol/L). Average blood ketones were highest during the food delivery phase (weeks 0–4). The highest average ketone levels were collected during week 3. Following the food delivery phase, average blood ketones slowly dropped as participants were self-providing food. By week 11 and 12 blood ketone measurements returned to levels observed at the beginning of the WFKD phase. Average β-Hydroxybutyrate measurements at the beginning of the diet phase, week 4, and week 12 closely matched mean measurements from the participants’ reported finger sticks. The values were below ketosis level at the beginning of the WFKD (0.12 mmol/L), peaked at 1.1 mmol/L and decreased to 0.51 mmol/L by week 12 of the WFKD phase. 

### 3.6. Satisfaction with Study Diets

**Figure 4:**
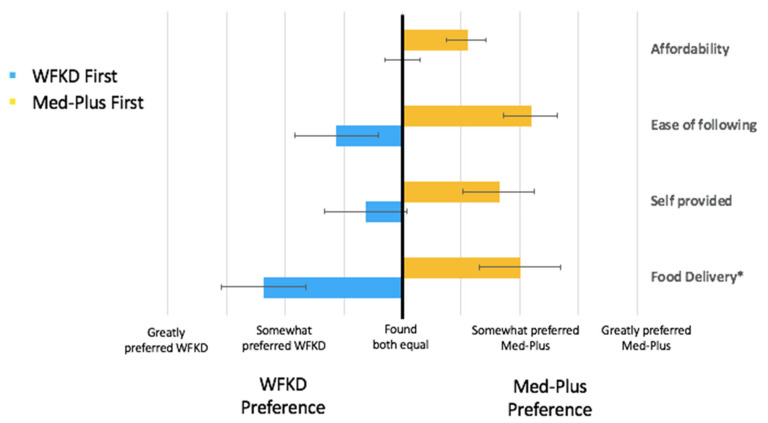
Mean and standard deviation of food satisfaction between Med-Plus and WFKD depending on what diet participants were assigned first. * For the food delivery, *n* = 6 receiving WFKD as the second phase and *n* = 7 receiving Med-Plus as the second phase did not receive food delivery due to COVID-19 disruption, as previously described.

When comparing both diets after completing both phases, a clear trend was observed with respect to diet preference—participants preferred the diet they were assigned to first (Figure 4). The trend is seen in all diet aspects, including affordability, ease of following, and dietary enjoyment during both the food delivery and self-provided periods. 

**Figure 5:**
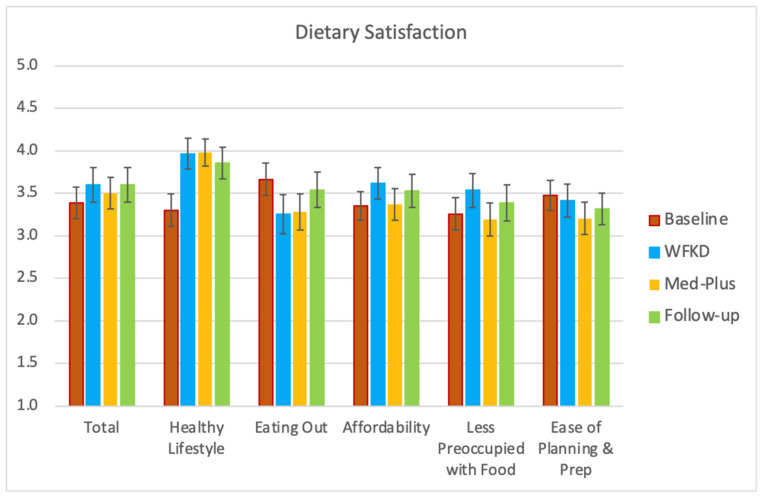
Mean and standard error of participant reported diet satisfaction at four Keto–Med Randomized Trial time points: baseline, end of diet 1, end of diet 2, and follow-up using the Diet Satisfaction Questionnaire (DSat-28©).

Total dietary satisfaction did not significantly change while on the assigned diets compared to their baseline diets. However, participants did believe that they were leading a healthier lifestyle on both study diets as well as in follow-up relative to baseline (Figure 5). Participants ate out less during the study and did not believe that the cost of either diet differed greatly from their usual diets. Neither preoccupation with food nor the time spent for planning and preparing meals increased during either of the study diet phases. 

### 3.7. Qualitative Comments about Study Diets

When asked for comments about preferences for either diet, the comments were equally split for WKFD and Med-Plus (Supplemental Table S5). Many of the comments had to do with specific food items that participants preferred including (e.g., cream in coffee, cheese, pork, fruit, grains) or preferred avoiding (e.g., fish, fat). Other comments had to do with cost, flexibility, social aspects of eating, ease of preparation or ordering in restaurants.

### 3.8. Assessment of COVID-19 Related Alterations

Overall, participants who responded to a survey did not believe that the shelter-in-place order affected their ability to adhere to their assigned diets, as only five indicated <100% adherence (with values ranging from 60–80%) (Supplemental Figure S5A–C). There were 23 of 40 participants whose participation was affected by the COVID-19 disruption, and 14 of the 23 responded to the survey; we were unable to obtain survey responses from 9 of the 23 despite repeated attempts. Of those impacted, most indicated being only somewhat rather than greatly impacted, and food accessibility was a greater factor than other COVID-19 related stressors. Most participants either decreased or did not change their level of physical activity, while very few participants increased their physical activity level. Of those who did not change the amount, a subset changed the type of physical activity they were doing prior to COVID-19. 

### 3.9. Adverse Events

During the trial, there were no serious adverse events requiring hospitalization. There was one adverse event related to the study or possibly related (a high alanine aminotransferase (ALT) lab value during the WFKD phase, which was within normal limits when rechecked three weeks later).

## 4. Discussion

In this randomized cross-over study contrasting a Ketogenic vs. Mediterranean diet among adults with either T2DM or prediabetes, we determined that average adherence between the two diet patterns was very similar during the intervention and involved substantial changes from prestudy dietary patterns. As might be expected, average adherence was best during the first four weeks of the 12-week diet phases when all meals were provided by a food delivery service. When study participants were responsible for purchasing and preparing their own meals, average adherence was then lower, but still significantly different and higher than prestudy diets. Importantly, for the internal validity of the study, the adherence scores were strikingly similar between WFKD and Med-Plus at both the 4-week and 12-week intervention time points. Throughout the study, a wide range of adherence variability was observed among participants—this was true for both diet types, and for both the food delivery phase and the self-provided food phase. The primary goal of the main study was to contrast the impact on blood glucose control of the two study diets, which is being reported in a separate manuscript. The primary objective of this secondary analysis was to quantitatively and qualitatively examine how well study participants understood the diets, how well they were able to adhere to those two diets, and the degree to which equipoise was achieved. We believe this is an important and often missed step in the analysis and interpretation of outcomes from dietary pattern intervention studies.

Given the tailored definitions for the WFKD and Med-Plus in this study, quantifying adherence required the development of novel metrics. While there are extensive studies that have reported adherence scores for a Mediterranean diet, most of these are observational cohort studies and there is no single scoring metric for which consensus has been reached regarding a standard definition. Previously used definitions are typically complex and are varied in the number of factors (foods, food groupings, and beverages) specified for adherence ranging from 8–15 [28,29,30,31,32,33]. For the Ketogenic diet, the situation is somewhat different. There have been many intervention studies published that generally use the same criteria for adherence, the focus being almost solely on the restriction of carbohydrates. However, there is still some variability in terms of grams vs. percent of carbohydrates, the specific cut points selected for those grams or percentages, and whether this refers to total carbohydrates or net carbohydrates [34,35,36,37,38]. Additionally, some sources have a differential target for initial vs. long-term carbohydrate intake.

Notably, many of the reported trials testing Ketogenic or Mediterranean diet patterns do not present information on adherence [39,40,41,42,43]. Additionally, some studies included a control comparison diet, but do not provide data on adherence to the control diet [44,45,46]. While several examples of adherence assessment were found among identified intervention trials with free-living participants, the general adherence was not particularly strong for either Ketogenic [47,48,49,50] or Mediterranean [51] diet patterns. An adherence paper was reported for the DIRECT Study, which asked participants to rate their own adherence to their assigned diet on a scale from 0–100 [52]. Although there are likely limitations with the accuracy of the self-reported adherence, the results indicated lower adherence for a low-carb diet after 2 years (78%) compared to the low-fat (90%) and the Mediterranean diet (85%) (*p* = 0.042 between groups) [52]. While follow-up in the DIRECT Study was longer than in this study, results are similar in that participants had greater difficulty adhering to the WFKD at follow-up.

For the current study, previous definitions of adherence to the two diet patterns were not well aligned with the tailored approach that we were testing. Our definition of the Mediterranean diet was stricter than others due to the emphasis on limiting added sugars and refined grains. For both diet patterns, the inclusion of nonstarchy vegetables was a characteristic weighted more heavily in our adherence score than others. Thus, novel adherence scores were developed specifically for this study as described in the methods section. While this limits the opportunity to compare our findings of adherence to those in other studies with different scoring criteria, the multiple characteristics included in the scores and the weighted point values assigned to each characteristic closely reflect the instructions and recommendations provided to participants in this study. 

Adherence in nutrition intervention diet pattern studies differs inherently by type of study. Feeding studies conducted with in-patient or domiciled study participants provide the greatest opportunity for perfect or near-perfect adherence. Strong examples of this come from the Hall research group [53,54,55]. While this allows for optimal dietary adherence, these types of studies are very expensive and are typically restricted to small sample sizes for short time periods. Thus, an important adherence issue regarding feeding studies is the tradeoff between rigor and generalizability [17]. While rigor is a strength, a criticism of feeding studies is a lack of generalizability of findings regarding feasibility and sustainability. 

In contrast, diet pattern studies that are done in free-living individuals who are provided with instructions and support are likely to have greater generalizability but inherently lower adherence levels due to lack of control over what participants consume. The approach used in the design of the current study was to try to balance rigor and generalizability. To maximize adherence in the free-living population of this study, all food was provided for the first 4 weeks of each diet phase. This provided participants the opportunity to be maximally adherent to the diet starting from the first day of initiation of each of the two diets, although there were still opportunities for deviations since the participants were not domiciled and monitored at all times. During the food delivery phase the health educators were able to explain the diet details to participants in very practical terms, working with them on challenges resulting from adjusting to an assigned diet, and helping them to develop plans for shopping and cooking for themselves during the self-provided portion for that same diet. As reported, adherence was high during the food delivery phase for both the WFKD and Med-Plus phases and very similar quantitatively (~7.5/10) in terms of the adherence scores that were standardized to a range of 0-10.

We initially assumed that study participants would be reluctant to shift to purchasing and preparing their own foods after receiving free delivery of foods. Thus, we provided an option to continue receiving some of the delivered foods at the participant’s own cost. We observed, somewhat to our surprise, that after 4 weeks of food delivery, all of the participants declined and preferred to prepare their own foods, largely due to an interest in a greater variety of foods than had been provided in the seven-day cycle of delivered foods for the past month. Although average adherence decreased during the self-provided phase, there were a few exceptions where adherence to either diet increased for a select number of participants. This may have been due to greater flexibility in being able to select preferred foods that still met macronutrient targets rather than foods provided by the delivery service. For others, having access to greater variety was welcomed but also provided more opportunities to deviate from the prescribed diet pattern parameters. At this stage all participants were eager to receive shopping lists, recipes, and sample menus—which were provided—and most participants expressed strong intentions of using these materials. Interestingly, we observed that these were seldom used. Participants ended up making modest but not major modifications to their habitual choices. Although disappointing, this speaks importantly to the feasibility and long-term sustainability of diet patterns, as participants by that time were well informed as to the specifics of following the diet from the examples of the delivered food and the repeated sessions with the study health educators.

A notable issue encountered by the health educators on both diets was variability in the participant’s own definition of success. For the study team, success meant strict adherence to the prescribed diet. However, for many participants, the goal was to lose weight, limit food intake during certain hours or be able to continue to consume their favorite foods while minimizing glucose excursions—none of which were actual objectives of the prescribed diet intervention. It was observed that when participants did not experience fulfillment of their personal goals, they were discouraged from following the diet. In an attempt to address this, participants were encouraged to log and share their food choices daily using Cronometer to help educators review their diets and give specific recommendations. Educators were sensitive to participants’ preferences and goals and worked with each one individually to find a realistic way to adhere to the diet as much as possible given their particular situation. Although this deviated substantially from perfect adherence, this was a reflection of what clinicians are likely to encounter when working with patients on achieving and maintaining dietary changes and therefore emphasized generalizability, as intended.

There were a few distinct issues specific to each diet that created relative challenges and advantages regarding adherence. A relative challenge to the Med-Plus diet was that the guidelines were more complex than WKFD. While on the Med-Plus phase, participants were instructed to find ways to incorporate multiple servings of legumes, fruits, and whole grains, preferably every day. This also required taking into consideration the many different types of legumes, fruits, and whole grains available, and finding recipes or menu items that were personally appealing. Participants often expressed to the health educators that they found it difficult to know if they were appropriately following the Med-Plus diet. 

In contrast, the WFKD was a very simple diet to explain and understand, and it was easy for participants to follow the instructions—for legumes, fruits, and grains, none were allowed (with the exception of occasional berries). Another adherence advantage for the WFKD was the home-based measurement of ketone levels which provided feedback on being in ketosis, whereas there was no parallel biochemical parameter being assessed for Med-Plus adherence. However, a challenge during the WFKD phase was the extreme carbohydrate restriction, which meant the exclusion of many commonly consumed foods, making it harder to follow in the long-term. This exclusion of foods made it more challenging for participants that shared meals with their families, with many participants finding that they often had to make separate meals for themselves and their families while on the WFKD. 

The use of CGMs at weeks 4 and 12 of the diet phases was a beneficial factor that helped with adherence for both diets. These devices provided real-time information on blood glucose response to the meals and snacks consumed. Many participants reported that seeing these values was very educational and contributed to more successful behavior change. 

The COVID-19 pandemic with the shelter-in-place ordinance started when approximately half of the participants were still going through the protocol. Thirteen of the 40 participants did not get the provided food delivery meals for the second of the two diet phases. Some participants had to extend the intervention period of one of the two diet phases, as long as two extra months, and some completed virtual meetings with health educators rather than in-person meetings. During that time, the health educators made sure to contact participants using their preferred mode of communication (Zoom, phone, and/or email) and worked with them on the immediate challenges, such as access to appropriate food, while also allowing them to express their emotional distress. For many, being in the study was a helpful way of dealing with the many changes to their lives as it gave them a concrete goal and motivation to continue to nourish themselves in a healthy manner. An important insight gleaned was that the frequency of the meetings was more important than the length of the visit. The brief Zoom calls and emails compared to in-person also served to reduce the participant burden of having to come to in-person meetings.

This study has several strengths including the crossover design which allowed each participant to serve as their own control. Each diet phase of the study included two stages—food delivery and self-provided, helping to maximize the overall rigor and generalizability of the study, respectively. A 12-week follow-up phase provided insights about sustainability—long-term dietary behaviors after participants completed the study and were free to choose which diet pattern they wanted to follow. Dietary adherence was determined using two measures of 24-h dietary assessment: dietary recalls collected at specific time points by trained study staff (via NDS-R) and food logging that could be completed at any time throughout the study by participants (Cronometer). In addition to dietary data, for the WFKD phase participants measured their blood ketone levels which provided a biological method of adherence to which we were able to compare our more subjective WFKD adherence score. 

The study also involved several limitations. First, due to the COVID-19 pandemic and shelter-in-place restrictions, many participants were on the study diets for longer than intended per protocol. This contributed to four study drop-outs. Despite the unexpected circumstances, the study had overall high retention and minimal missing data. The missingness of the data during shelter-in-place was minimized by the ability to conduct surveys and collect CGM data and diet assessment data remotely. It is important to note that our adherence scoring for both diets was novel and was not validated; however, a review of the literature suggests there is no consensus on a single adherence definition for either the Ketogenic or the Mediterranean diet. We also had a team of registered dietitians review adherence scoring for content validity. Further, although the use of multiple measures of dietary assessment is a strength, the self-reported diet data from both methods remains prone to reporting bias and measurement error. This limitation was also somewhat minimized for the first 4 weeks of each diet phase when selected diet-pattern compliant foods were delivered, allowing for increased knowledge of what participants should have been consuming and reporting.

## 5. Conclusions

For nutrition intervention studies involving dietary patterns, it is obviously important to have participants follow protocol diets as closely as possible, and to measure actual adherence assessments in order to inform the interpretation of main findings [17]. Unfortunately, we find that this is seldom done. Macronutrient content is only one of many factors influencing adherence. Dietary choices and behaviors are complex and determined by different individual-level factors (e.g., food preference, knowledge), cultural factors (e.g., social support and social norms), economic factors (e.g., income), and the nutrition environment (e.g., access, availability) [56]. Ideas to increase adherence include supplying food, close monitoring, clear diet instructions, opportunities for participants to measure their own adherence (e.g., ketones, tallying carbs, CGMs), personalized advice and recommendations acknowledging participants efforts and goals, working with participants through life challenges experienced during the study protocols, and framing study adherence as a way of having some control of their lives. This in-depth assessment of adherence will serve to help with the interpretation of the main study findings that will be reported separately. Finally, we hope the approaches taken in this secondary analysis of adherence will help to inform other investigators of strategies and assessments they may want to incorporate in future nutrition intervention studies.

## Figures and Tables

**Table 1:** Key Similarities and Differences for the Two Keto–Med Randomized Trial Diet Patterns.

Carbohydrate Sources	Keto	Med
	**Similar**	
Added sugars	Avoid	Avoid
Refined grains	Avoid	Avoid
Nonstarchy vegetables	Include	Include
	**Different**	
Legumes	Avoid	Include
Fruits	Avoid	Include
Whole grains	Avoid	Include

**Table 2:** Keto–Med Randomized Trial Participants’ Baseline Sociodemographic, Anthropometric, and Metabolic Characteristics ^1^.

	Keto–Med (*n* = 20)	Med–Keto (*n* = 20)	All (*n* = 40)
**Gender, *n***			
Male	9	9	18
Female	11	11	22
**Diagnosis, *n***			
Prediabetes	10	11	21
Type 2 Diabetes Mellitus	10	9	19
**Age, year**	57.8 ± 9.2	59.6 ± 8.8	58.7 ± 9.1
**Highest level of education achieved, *n***			
High school graduate	1	2	3
Some college	2	2	4
College degree	6	6	12
Some postgraduate school	3	2	5
Postgraduate degree	8	8	16
**Race/ethnicity, *n***			
Non-Hispanic White	11	8	19
Hispanic/Latinx	2	5	7
Asian	6	5	11
Black/African American	0	1	1
Native Hawaiian/Pacific Islander	1	1	2
**Weight, kg**			
Male	87.3 ± 12.1	88.5 ± 17.4	87.9 ± 14.1
Female	92.8 ± 13.3	85.0 ± 16.6	88.9 ± 14.8
Both sexes	90.3 ± 12.7	86.6 ± 16.6	88.4 ± 14.5
**Body Mass Index (kg/m^2^)**			
Male	28.1 ± 3.5	29.6 ± 4.6	28.9 ± 3.9
Female	33.7 ± 3.5	32.2 ± 5.6	33.0 ± 3.5
Both sexes	31.2 ± 4.4	31.1 ± 5.2	31.1 ± 4.7
**Blood pressure, mmHg**			
Systolic	122.6 ± 11.3	123.1 ± 13.9	122.8 ± 12.4
Diastolic	76.0 ± 10.0	74.5 ± 8.3	75.2 ± 9.0
**Blood lipids, mg/dL**			
HDL cholesterol	48 ± 11	46 ± 10	47 ± 10
LDL cholesterol	100 ± 31	107 ± 34	104 ± 32
Triglycerides	150 ± 97	132 ± 65	141 ± 81
**Fasting glucose, mg/dL**			
Persons with prediabetes	111.2 ± 6.4	103.4 ± 12.0	107.1 ± 10.5
Persons with Type 2 Diabetes Mellitus	138.4 ± 26.1	143.8 ± 31.3	140.9 ± 28.8
**Fasting insulin, µIU/mL**			
Persons with prediabetes	21.6 ± 14.4	21.5 ± 14.5	21.6 ± 14.4
Persons with Type 2 Diabetes Mellitus	20.9 ± 9.3	12.4 ± 4.4	16.9 ± 8.5
**HbA1c levels, %**			
Persons with prediabetes	5.8 ± 0.3	5.8 ± 0.3	5.8 ± 0.3
Persons with Type 2 Diabetes Mellitus	7.0 ± 0.7	6.9 ± 0.8	6.9 ± 0.8
**Alanine aminotransferase (ALT), IU/L**	29 ± 13	27 ± 12	28 ± 12

^1^ Values are *ns* or means ± SDs.

**Table 3:** Macronutrients of Foods as Designed, as Delivered, and as Consumed During Food Delivery Component of the Keto–Med Randomized Trial.

	Kcals	% Fat	% Carbohydrates	% Protein	% Alcohol
**As Designed** ^1^					
WFKD	2796	69	7	24	--
Med-Plus	2784	48	36	16	--
**As Delivered** ^2^					
WFKD	2816	66	5	29	--
Med-Plus	2952	46	38	16	--
**As Consumed** ^3^					
WFKD	1479 ± 420	63 ± 7.5	10 ± 4.5	25 ± 6.5	2 ± 3.1
Med-Plus	1562 ± 399	45 ± 6.9	34 ± 8.8	19 ± 6.3	2 ± 3.5

Abbreviations: WFKD, Well Formulated Ketogenic Diet; Med-Plus, Mediterranean Plus Diet. ^1^ Diets as designed by the research team. ^2^ Foods that were able to be produced and delivered by food delivery company (Methodology). ^3^ Average of participant intake during the diet phase. “As consumed” averages may be different than “as delivered” as participants were given a week’s supply of meals, encouraged to eat a particular proportion of each menu item over the course of the week, but ultimately may have had uneven proportions of menu items over the course of the week. Further, no alcohol was included in the designed or delivered items, but some participants reported modest amounts of alcohol consumption during the “food delivery” phase.

**Table 4:** Comparison of Adherence Scores between Keto–Med Randomized Trial Diet Phases by Timepoint (maximum adherence score = 10).

Timepoint	Well Formulated Ketogenic Diet		Mediterranean Plus		Between Diet *p*-Value ^1^
	Mean ± SD	Range	Mean ± SD	Range	
Food Delivery	7.6 ± 2.1	1.2–10.0	7.3 ± 1.5	3.5–9.6	0.333
Self-Provided Food	5.7 ± 3.0	0.3–9.7	5.4 ± 1.5	1.7–8.3	0.641

^1^ Matched-Pairs *t*-Test of between diets.

## Data Availability

The data that support the findings of this study are available from the corresponding author (C.D.G.), upon reasonable request.

## Supplementary Materials

The following are available online at https://www.mdpi.com/2072-6643/13/3/967/s1, Figure S1: Keto–Med Randomized Trial Study Design; Table S1: Foods Provided During the Food Delivery Component of the Keto–Med Randomized Trial; Table S2: Well Formulated Ketogenic Diet (WFKD) Scoring; Table S3: Mediterranean Diet Plus (Med-Plus) Scoring; Figure S2: Consort Diagram for the Keto–Med Randomized Trial; Figure S3A–E: Nutrient Data by Randomization Order; Table S4: Comparison of Adherence Scores Within Keto–Med Randomized Trial Diet Phases by Timepoint; Figure S4: Average Blood Ketones Per Week During WFKD Phase; Table S5: Selected Qualitative Responses from Participants about Likes/Dislikes of Study Diets; Figure S5A–C: COVID-19 Related Alterations on Adherence and Physical Activity. Supplemental File S1: Modifications to the Study Design Due to COVID-19; Supplemental File S2: WFKD Diet Instructions; Supplemental File S3: Med-Plus Diet Instructions; Supplemental File S4: Instructions to Start Cooking WFKD; Supplemental File S5: Instructions to Start Cooking Med-Plus; Supplemental File S6: Keto–Med Randomized Trial Questionnaire Items for Food Satisfaction, Diet Satisfaction, and COVID-19 Related Alterations
