# Supplementary material for: Adherence to Ketogenic and Mediterranean Study Diets in a Crossover Trial: The Keto–Med Randomized Trial
Source: Nutrients. 2021 Mar 17;13(3):967. doi: 10.3390/nu13030967 (PMC8002540; doi:10.3390/nu13030967)
Supplement: Supplementary file 1 [file nutrients-13-00967-s001.zip › Keto-Med Adherence Supplement.docx]

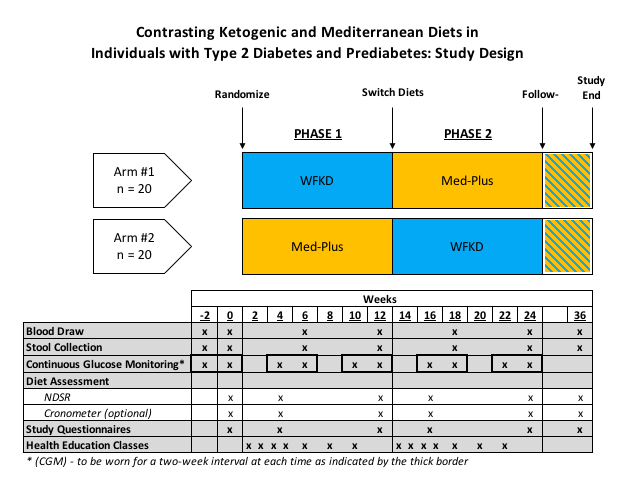


**Figure S1**. Keto-Med Randomized Trial Study Design

| **Table S1.** Foods Provided During the Food Delivery Component of the Keto-Med Randomized Trial | |
| --- | --- |
| **Well Formulated Ketogenic Diet (WFKD) Foods** | **Mediterranean Plus Diet (Med-Plus) Foods** |
| Scrambled Eggs with Ratatouille | Vanilla Overnight Oats |
| Scrambled Eggs with Pork Chops | Strawberry Mango Overnight Oats |
| Pastured Pulled Pork | Blueberries |
| Seared Flank Steak | Strawberries |
| Pan Seared Prawns | Scrambled Eggs with Sesame Mushrooms |
| Roasted Salmon | Lemon Pepper Chicken |
| Smoked Salmon | Blackened Cod |
| Pork Chorizo | Wild Petrale Sole |
| Braised Tuscan Kale & Heirloom Cherry Tomatoes | Braised Tuscan Kale & Heirloom Cherry Tomatoes |
| Slaw Salad | Ratatouille |
| Broccolini | Broccolini with Roasted Garlic |
| Spinach | Slaw Salad |
| Celery | Pea Shoots |
| Roasted Artichoke Hearts | Quinoa Tabbouleh Salad |
| Cilantro Lime Crema | Whipped Sweet Potatoes |
| Avocados | Cannellini Beans with Herb Puree |
| Olive oil | Lemon Leek Chickpeas |
| Ghee | Crunchy Chickpeas |
| Chocolate Raspberry Keto Fat Bombs | Trail Mix Topping |
|  | Avocado |
|  | Hemp Seeds |
|  | Cilantro Lime Dressing |
|  | Olive Oil |

| **Table S2**. Well Formulated Ketogenic Diet (WFKD) Scoring^1^ | | | | | | | | |
| --- | --- | --- | --- | --- | --- | --- | --- | --- |
| **Component** | **Units** | **Points** | | | | | **Score Weight** | **Scoring Factor** |
|  |  | 0 | 1 | 2 | 3 | 4 |  |  |
| Net Carbohydrates^2^ | grams | ≥60 | 51-59 | 41-50 | 31-40 | ≤30 | 50% | 1.25 |
| % Calories from Fat | percent | ≤50 | 51-55 | 56-60 | 61-65 | ≥65 | 12.5% | 0.31 |
| Non-Starchy Vegetables | servings | <1.0 | 1.0-1.9 | 2.0-2.9 | 3.0-3.9 | ≥4.0 | 12.5% | 0.31 |
| Added Sugar | grams | ≥4.0 | 3.0-3.9 | 2.0-2.9 | 1.0-1.9 | 0-0.9 | 12.5% | 0.31 |
| Refined Grains | ounce equiv. | ≥1.0 | 0.75-0.9 | 0.5-0.74 | 0.25-0.49 | 0-0.24 | 12.5% | 0.31 |
| ^1^A total score was derived from multiplying the scoring factor by the point value for each component then summing up the 5 component scores. Maximum score of 10.  ^2^Net Carbs was derived by subtracting total dietary fiber from total carbohydrates. | | | | | | | | |

| **Table S3**. Mediterranean Diet Plus (Med-Plus) Scoring^1^ | | | | | | | | |  |
| --- | --- | --- | --- | --- | --- | --- | --- | --- | --- |
| **Component** | **Units** | **Points** | | | | **Score Weight** | **Scoring Factor** | | |
|  |  | 0 | 1 | 2 | 3 | 4 |  |  | |
| Non-Starchy Vegetables | servings | ≤0.4 | 0.5-1.5 | 1.6-3.0 | 3.1-6.4 | ≥6.5 | 16.6% | 0.415 | |
| Intact Whole Grains and Starchy Vegetables | servings | ≤0.4 | 0.4-0.7 | 0.8-1.5 | 1.6-2.9 | ≥3.0 | 16.6% | 0.415 | |
| Added Sugar | grams | ≥20.0 | 15-19 | 10-14 | 5-9 | ≤4.0 | 16.6% | 0.415 | |
| Refined Grains | ounce equiv. | ≥1.5 | 1.1-1.4 | 0.7-1.0 | 0.3-0.6 | ≤0.2 | 16.6% | 0.415 | |
| Fruits | servings | ≤0.4 | 0.5-0.9 | 1.0-1.4 | 1.5-1.9 | ≥2.0 | 8.3% | 0.208 | |
| Legumes | servings | ≤0.3 | 0.4-0.7 | 0.8-1.5 | 1.6-2.9 | ≥3.0 | 8.3% | 0.208 | |
| Fish | ounces | ≤0.2 | 0.3-1.5 | 1.6-2.5 | 2.6-3.9 | ≥4.0 | 8.3% | 0.208 | |
| Red Meat (Beef, Pork, Lamb) | ounces | ≥2.0 | 1.5-1.9 | 1.0-1.4 | 0.5-0.9 | ≤0.4 | 8.3% | 0.208 | |
| ^1^ A total score was derived from multiplying the scoring factor by the point value for each component then summing up the 8 component scores. Maximum score of 10. | | | | | | | | |  |

**Figure S2**. Consort Diagram for the Keto-Med Randomized Trial

| 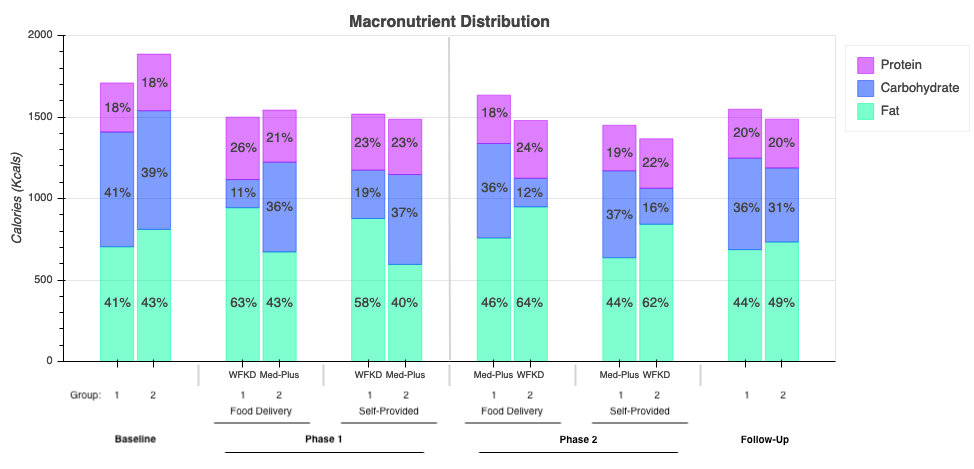  **A** | |
| --- | --- |
| 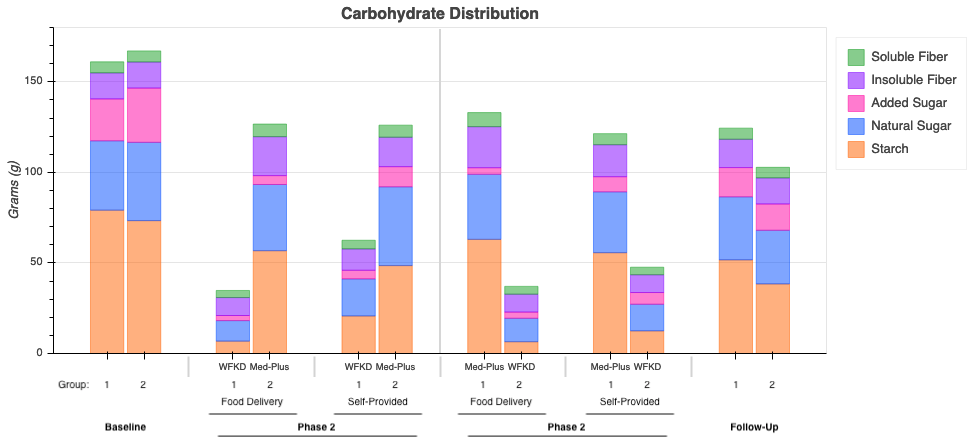  **B** | 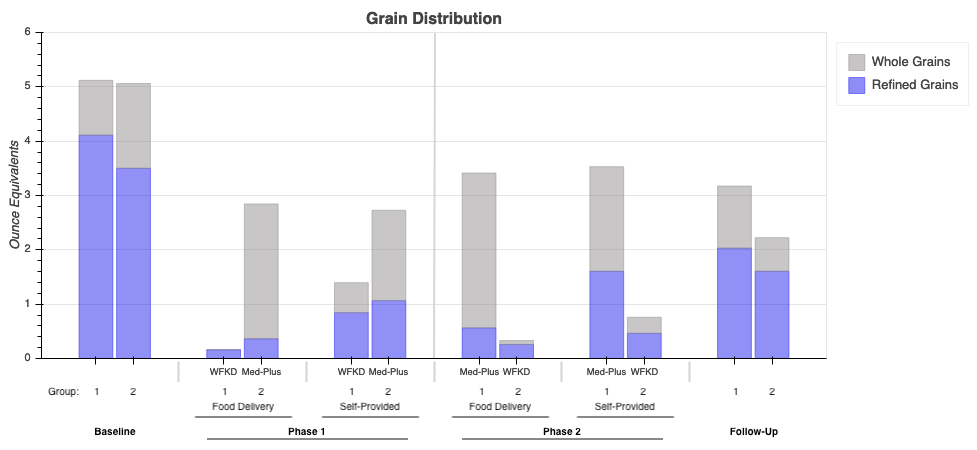  **C** |
| 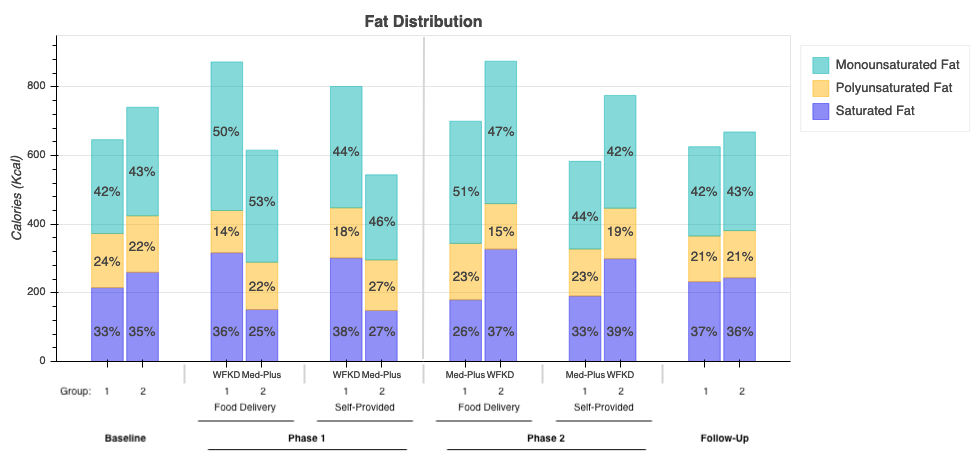  **D** | 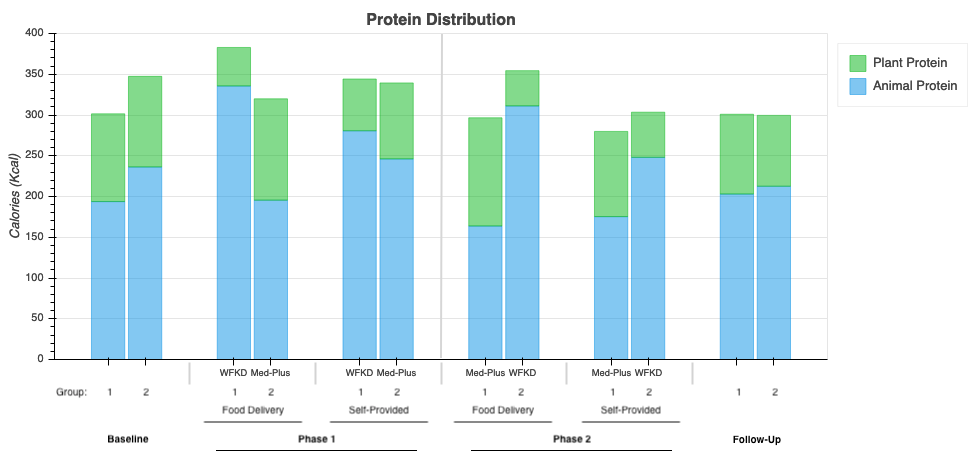  **E** |

**Figure S3A-E: Nutrient Data by Randomization Order.** Nutrient data for means of (A) macronutrients, (B) carbohydrates, (C) whole and refined grains, (D) fats, and (E) Animal and plant proteins at 6 time points (baseline, food delivery and self-provided during diet during phase 1, food delivery and self-provided during diet phase 2, and follow-up) by randomization order (denoted by group number). Group 1 received the WFKD during the first phase and Med-Plus during the second phase, vice-versa for Group 2.

| **Table S4:** Comparison of Adherence Scores Within Keto-Med Randomized Trial Diet Phases by Timepoint | | | | | |
| --- | --- | --- | --- | --- | --- |
|  |  | **Matched-Pairs t-Test p-Value of Within Diet Phase** | | | |
|  | **Timepoint** | Baseline | Food Delivery | Self-Provided | Follow-Up |
| **Well Formulated Ketogenic Diet** | Baseline | ---- |  |  |  |
|  | Food Delivery | <0.001 | ---- |  |  |
|  | Self-Provided Food | <0.001 | 0.001 | ---- |  |
|  | Follow-Up | 0.002 | <0.001 | <0.001 | ---- |
|  |  | **Matched-Pairs t-Test P-Value of Within Diet Phase** | | | |
|  | **Timepoint** | Baseline | Food Delivery | Self-Provided | Follow-Up |
| **Mediterranean Plus** | Baseline | ---- |  |  |  |
|  | Food Delivery | <0.001 | ---- |  |  |
|  | Self-Provided Food | <0.001 | <0.001 | ---- |  |
|  | Follow-Up | 0.003 | <0.001 | <0.001 | ---- |

**Figure S4: Average Blood Ketones Per Week During WFKD Phase** Individual participant change in blood ketone measurements during the food delivery and self-provided phases of the WFKD. Points represent participants’ weekly average of blood ketones. Solid black line represents the mean. Blue shaded area (between 0.5 and 3 on the y-axis) represents the cutoffs for nutritional ketosis. Yellow triangles represent the average β-Hydroxybutyrate measurements (via venipuncture) at three timepoints (beginning of the WFKD phase, week 4 of WFKD, and week 12 of WFKD).

| **Table S5**: Selected Qualitative Responses from Participants about Likes/Dislikes of Study Diets | |
| --- | --- |
| **Preferred WFKD** | **Preferred Med-Plus** |
| Does not like fish | Less expensive |
| Easy to follow | More flexible |
| Easy to order in restaurants | Wine |
| Cream in coffee | Did not feel well on the keto diet, no energy |
| Cheese, pork | Did not like eating that much fat |
| Others in household brought sweets and other foods and it made it hard | If I take a break from the keto, I won’t be able to go back to it |
| Very easy to cook for this diet | Miss fruit and grains on keto |
| Med diet was harder to understand | The whole family can eat together |
| Liked the blood glucose levels | Keto as a weight loss diet, med for long term |
| Volume of food on Med diet is much larger |  |

| **A** | |
| --- | --- |
| **B** | **C** |

**Figure S5A-C: COVID-19 Related Alterations on Adherence and Physical Activity.** Participant’s perceptions of COVID-19’s impact on A) adherence to the study protocol B) factors influencing adherence, and C) physical activity (PA). Data are provided for 14 participants of 23 who were impacted by COVID-19 and responded to the survey.
