# Supplementary material for: Adherence to Ketogenic and Mediterranean Study Diets in a Crossover Trial: The Keto–Med Randomized Trial
Source: Nutrients. 2021 Mar 17;13(3):967. doi: 10.3390/nu13030967 (PMC8002540; doi:10.3390/nu13030967)
Supplement: Supplementary file 1 [file nutrients-13-00967-s001.zip › Supplemental File 1.docx]

**Keto-Med REDCap Project**

**README File**

**Table of Contents**

[Data Collection Events 2](#_Toc53652295)

[COVID-19 Protocol Modification – Summary 2](#_Toc53652296)

[REDCap Project Modifications 3](#_Toc53652297)

[Event Modification – Screenshot 3](#_Toc53652298)

[Extra Timepoints 3](#_Toc53652299)

[**4b: extended diet 1/restart diet 2 for ppt 8016 only** 3](#_Toc53652300)

[**5b-** 4](#_Toc53652301)

[**6b: extended diet 2/end of diet 2 for ppt 8016 only** 4](#_Toc53652302)

[**7b: extended washout** 4](#_Toc53652303)

[Missed Timepoints 4](#_Toc53652304)

[Blood Draws from June 2020-onward 5](#_Toc53652305)

[Tracking COVID-19 impact in REDCap 5](#_Toc53652306)

[Visual depiction of the impact of COVID on each participant 6](#_Toc53652307)

#

# Data Collection Events

The Keto-Med study had 7 blood draw/data collection events, each represented by a Visit as follows:

**Visit 1-** Pre-baseline, eligibility check

**Visit 2-** Baseline, within 3 days of starting Diet 1

**Visit 3-** 4 weeks into Diet 1/end of Methodology for those who received it

**Visit 4-** 12 weeks/End of Diet 1

**Visit 5-** 16 weeks/4 weeks into Diet 2 & end of Methodology for those who received it

**Visit 6-** 24 weeks/12 weeks into & end of Diet 2

**Visit 7-** 36 weeks/Washout

The additional “Washout” column seen in the project layout (blacked out in the **Event Modification – Screenshot** image below) was originally part of the study as a 48 week timepoint but was removed prior to any participant reaching it.

# COVID-19 Protocol Modification – Summary

The COVID-19 pandemic started mid-way through the Keto-Med study and affected most of the study participants. Between mid-March to the end of May 2020, participants were not able to come in for a blood draw (hereafter referred to as “shutdown”) as residents of California and the Bay Area were mandated to shelter-in-place. Furthermore, the Clinical Translational Research Unit (CTRU) was unexpectedly closed down and open only to essential research appointments, of which the Keto-Med Study was not included. All activity at 1070 Arastradero was restricted as a result of the shelter-in-place & study supplies were mailed out rather than handed to participants at their appointments.

When COVID hit, 3 participants had completed the study entirely (including washout), 10 participants had completed both diets and were in washout, and 24 participants completed diet 1. There were 3 drops that occurred before COVID hit. See **Visual depiction of the impact of COVID on each participant** for full details.

Because we were unable to carry out all of the study tasks during this time, it was necessary to update the protocol to preserve the integrity of the study. This included making modifications to the REDCap project and adding extra data collection timepoints. This Readme document captures these changes.

# REDCap Project Modifications

## Event Modification – Screenshot


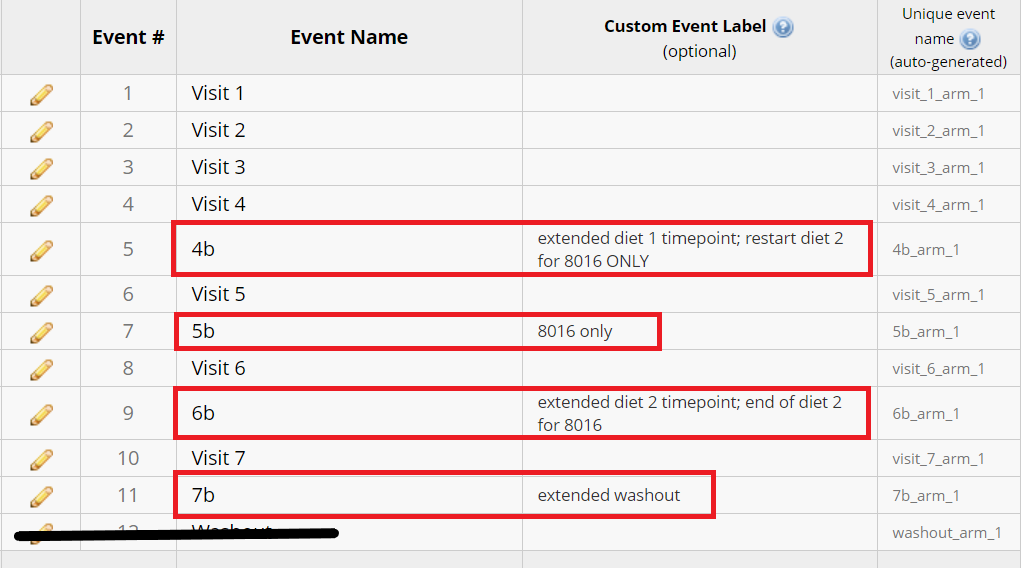


## Extra Timepoints

During the shutdown, extra timepoints were added for certain participants where, depending on their study Phase & appointment date, they were asked to collect additional stool samples, wear the CGM, perform NDSR recalls and/or fill in questionnaires. A description of each of these timepoints as well as the affected participants follows:

**4b “extended diet 1”/”restart diet 2 for ppt 8016 only”-** Participants with **Visit 4-end of diet 1** blood draws during the shutdown *that should have occurred more than 2 weeks before re-opening* were asked to collect an additional stool sample, wear a CGM & perform two additional NDSR recalls in the week prior to coming in for their rescheduled blood draw. The following participants were affected:

| **PPT** | **Additions** | | |
| --- | --- | --- | --- |
|  | **Stool** | **CGM** | **NDSR** |
| 8042 | 4b | 4b | 4b - 2 recalls only |
| 8045 |  |  |  |
| 8046 |  |  |  |
| 8048 |  |  |  |
| 8049 |  |  |  |
| 8050 |  |  |  |
| 8052 |  |  |  |

- For **ppt. 8016 only**: this is her “baseline” Diet 2 timepoint as she completed diet 1 with Visit 4 but did not restart diet 2 until 6 months later.

**5b-** only exists for **ppt. 8016** as she restarted Diet 2 but had already completed all of the data collection for Visit 5 during her first attempt at Diet 2. Data from this visit will be used for all analyses.

**6b “extended diet 2”/”end of diet 2 for ppt 8016 only”-** Participants with **Visit 6-end of diet 2** blood draws during the shutdown *that should have occurred more than 2 weeks before re-opening* were asked to collect an additional stool sample, wear a CGM & perform two additional NDSR recalls in the week prior to coming in for their rescheduled blood draw. The following participants were affected:

| **PPT** | **Additions** | | |
| --- | --- | --- | --- |
|  | **Stool** | **CGM** | **NDSR** |
| 8008 | 6b | 6b | 6b - 2 recalls only |
| 8014 |  |  |  |
| 8019 |  |  |  |
| 8029 |  |  |  |
| 8031 |  |  |  |
| 8034 |  |  |  |

- For **ppt. 8016 only:** this is her end of diet 2 timepoint that will be used for all analyses

**7b “extended washout”-** Participants with **Visit 7-washout** blood draws during the shutdown were asked to collect an additional stool sample in the week prior to coming in for their rescheduled blood draw. The following participants were affected:

| **PPT** | **Additions** | | |
| --- | --- | --- | --- |
|  | **Stool** | **CGM** | **NDSR** |
| 8001 | 7b | None | |
| 8003 |  |  |  |
| 8009 |  |  |  |
| 8012 |  |  |  |
| 8018 |  |  |  |
| 8022 |  |  |  |

## Missed Timepoints

The study team decided that participants whose Visit 3 or Visit 5 appointments fell during the shutdown would be cancelled and not rescheduled. The following participants had their appointments cancelled:

| 8047 | Visit 3 |
| --- | --- |
| 8049 | Visit 3 |
| 8053 | Visit 3 |
| 8056 | Visit 3 |
| 8060 | Visit 3 |
| 8061 | Visit 3 |
| 8062 | Visit 3 |
| 8063 | Visit 3 |
| 8064 | Visit 3 |

**Participants 8049, 8063 & 8064 dropped before reaching visit 4

| 8037 | Visit 5 |
| --- | --- |
| 8039 | Visit 5 |
| 8040 | Visit 5 |

In addition, the following participants missed their Visit 6 blood draws because they fell early in the shutdown and they were not willing to stay on their assigned diets until reopening, which was unknown at that time.

| 8023 | Visit 6 |
| --- | --- |
| 8029 | Visit 6 |
| 8031 | Visit 6 |

**Participant 8023 stayed on her assigned diet for 1 extra week before going into washout, ppt. 8029 for 6 extra weeks and ppt. 8031 for 5 extra weeks. For this reason, ppt. 8023 was not asked to complete additional timepoints as described above.

# Blood Draws from June 2020-onward

As a result of the CTRU closure, all remaining participants had to attend their blood draw appointments at the Crane Street Clinic in Menlo Park, which was a phlebotomy lab in the Stanford Healthcare Network. The study team prepared blood draw kits that were mailed to the participants and included blood draw tubes, an instruction sheet and a blood draw requisition form that they had to bring with them to their appointments. The Med-Speed Courier service was used to transport the samples from the clinic to the CTRU lab for processing within 2 hours of the blood draw. Because the clinic *did not* take vital measurements, we do not have clinical data (blood pressure, pulse, etc.) for anyone with appointments from June 2020 onwards. Participants were asked to measure their weight at home the morning of their appointment and record it in the Cronometer app if they did so.

# Tracking COVID-19 impact in REDCap

To document the impact that COVID-19 had on each participant, additional fields were added to the *Study Status* form. The number of **additional weeks** that a participant was in a particular phase was calculated & recorded in this form.


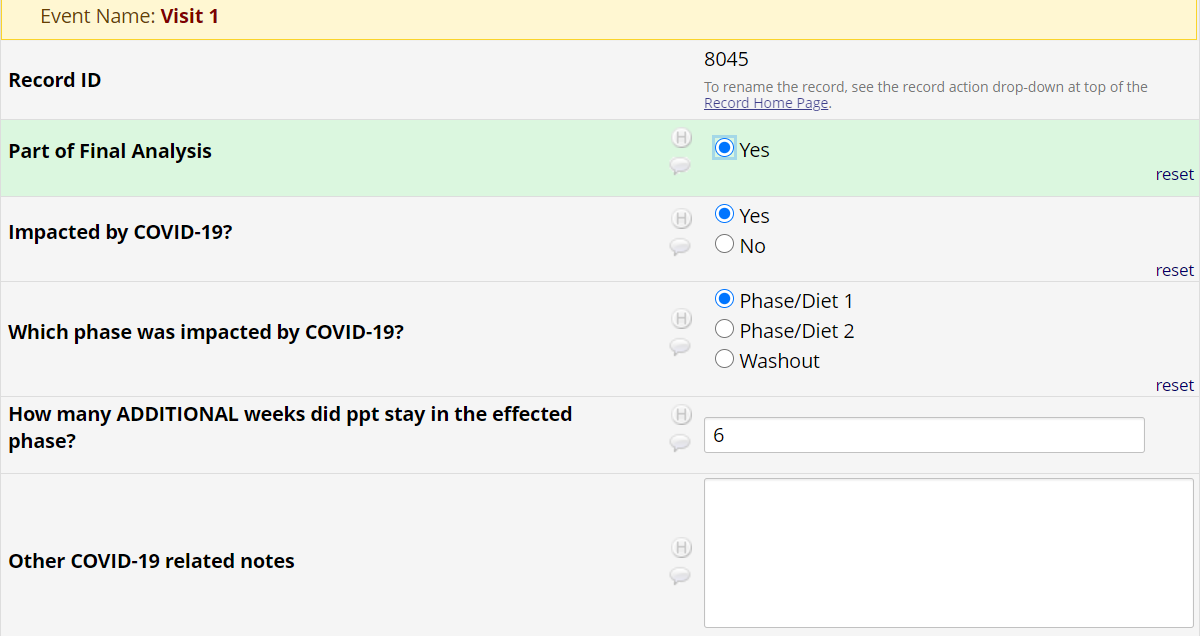


# Visual depiction of the impact of COVID on each participant:

| **Participant ID** | **Phase Pre-COVID** | **COVID Adjustments** |
| --- | --- | --- |
| 8001 | Washout | Visit 7 on hold |
| 8002 | Done | None |
| 8003 | Washout | Visit 7 on hold |
| 8005 | DROP | Dropped after finishing diet 1 |
| 8006 | Done | None |
| 8007 | Done | None |
| 8008 | Diet 2 | Diet 2 extended |
| 8009 | Washout | Visit 7 on hold |
| 8012 | Washout | Visit 7 on hold |
| 8014 | Diet 2 | Diet 2 extended |
| 8015 | Washout | Visit 7 on hold |
| 8016 | Diet 2 | Restarting Diet 2 |
| 8018 | Washout | Visit 7 on hold |
| 8019 | Diet 2 | Diet 2 extended |
| 8020 | Washout | Visit 7 on hold |
| 8021 | DROP | Dropped within days of starting study |
| 8022 | Washout | Visit 7 on hold |
| 8023 | Diet 2 | Missed Visit 6 appt. |
| 8024 | Washout | Visit 7 on hold |
| 8025 | DROP | Dropped within days of starting study |
| 8027 | Washout | Visit 7 on hold |
| 8029 | Diet 2 | Missed Visit 6 appt. – then DROPPED |
| 8031 | Diet 2 | Missed Visit 6 appt. |
| 8034 | Diet 2 | Diet 2 extended |
| 8037 | Diet 2 | Diet 2 extended |
| 8039 | Diet 2 | Diet 2 extended |
| 8040 | Diet 2 | Diet 2 extended |
| 8042 | Diet 1 | Diet 1 extended |
| 8045 | Diet 1 | Diet 1 extended |
| 8046 | Diet 1 | Diet 1 extended |
| 8047 | Diet 1 | Diet 1 extended |
| 8048 | Diet 1 | Diet 1 extended |
| 8049 | Diet 1 | Diet 1 extended – then DROPPED |
| 8050 | Diet 1 | Diet 1 extended |
| 8052 | Diet 1 | Diet 1 extended |
| 8053 | Diet 1 | Diet 1 extended |
| 8056 | Diet 1 | Diet 1 extended |
| 8060 | Diet 1 | Diet 1 extended |
| 8061 | Diet 1 | Diet 1 extended |
| 8062 | Diet 1 | Diet 1 extended |
| 8063 | Diet 1 | Diet 1 extended – then DROPPED |
| 8064 | Diet 1 | Diet 1 extended – then DROPPED |
