# Supplementary material for: Adherence to Ketogenic and Mediterranean Study Diets in a Crossover Trial: The Keto–Med Randomized Trial
Source: Nutrients. 2021 Mar 17;13(3):967. doi: 10.3390/nu13030967 (PMC8002540; doi:10.3390/nu13030967)
Supplement: Supplementary file 1 [file nutrients-13-00967-s001.zip › Supplemental File 2.docx]

**Ketogenic Diet**

**What is a ketogenic diet?**

A ketogenic diet is a diet that relies on ketones as a source of fuel for the body. When you eat carbohydrates, the preferred fuel for your body is glucose (sugar), but when you exclude carbohydrates from your diet, you use fats as a fuel. Breaking down fats creates ketone molecules, which are used in the cells of your body for fuel. To make this happen, the intake of carbohydrates has to be very low. You are encouraged to include as little carbohydrates and consume a high amount of fat. Excess protein can also be converted to glucose, so the diet should be only moderate in protein.

**What foods are included in the ketogenic diet?**

- Natural fats and oils: olive oil, canola oil, butter, coconut oil. Also good choices are avocado and olives.
- Meats: beef, lamb, pork, poultry, are all allowed. Higher fat choices are allowed, including the poultry skin.
- Eggs
- Fish and seafood: all allowed; fatty fish recommended.
- Dairy: cheese and cream. No milk or yogurt.
- Vegetables: limit to those that grow above the ground (except corn and peas)


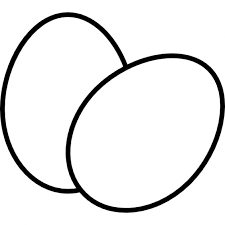

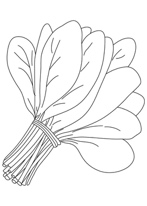

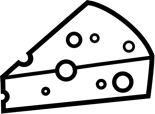

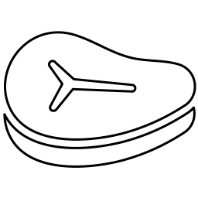

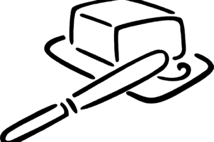


It is important to include a high percentage of your calories as fat. This means, use more oil or butter when cooking, add cream to your sauces, add avocados and olives to your salads, etc.

**What foods are not included in the ketogenic diet?**

The lower the amount of carbohydrate consumed, the more the body will rely on ketones for fuel. Following are the foods to avoid during the diet:

- Sodas and juices. Also eliminate sports drinks, “Vitamin water” and other beverages that contain sugar
- Chocolate and other candies
- Pastries of all kind
- Fruit: all fruit has carbohydrates. Small amounts of berries might be included.
- All starches, which include all the foods made from grains: breads, pasta, rice, oats, barley, buckwheat. Whole grain products are excluded as well.
- Vegetables that grow below the ground such as potatoes and yams. Some like beets and carrots might be included in very small amounts.
- Beans and lentils all are high in carbohydrates and should be avoided.

**
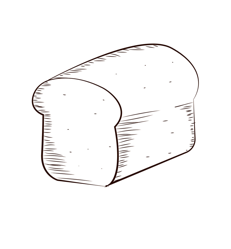

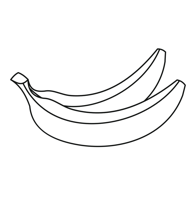

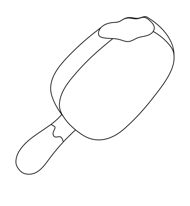

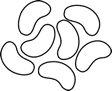

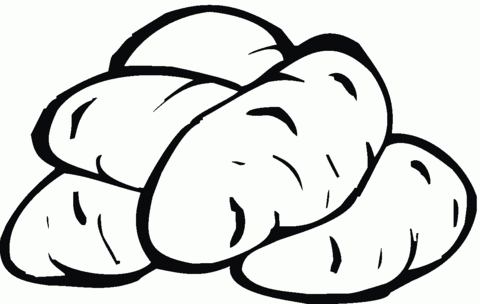
**

**What about alcohol?**

Your tolerance to alcohol is reduced on a ketogenic diet. You might get intoxicated with a lower intake. We recommend that you drink in moderation, no more than half of your usual intake.

- Low carbohydrate alcoholic drinks: dry red and white wines, champagne, straight spirits such as whiskey, dry martini, tequila shot, brandy.
- High carbohydrate alcoholic drinks: beer, most cocktails

**How do I follow this diet?**

The first 4 weeks, you will not have make many decisions, as you will be receiving your meals! During that period we will have opportunities to talk about shopping, cooking, eating out, etc. and you will feel ready to embrace this diet. We will have a combination of personal and virtual meetings to accommodate your needs and your schedule.

**What is included in my Methodology delivered meals?**

We worked very closely with the team at Methodology to develop the recipes for the foods you will receive. These meals follow the guidelines for a healthy, balanced ketogenic diet. Each component of a meal is delivered in a glass jar and you can mix and match the content to make the meal of your choice. Some examples of what you will get may include turkey chorizo and scrambled eggs, Portobello mushrooms and turmeric scrambled eggs, pistachio crusted cod, za’atar chicken breast, salmon cakes, asparagus, green beans, salads.

After the 4 weeks of delivered food, you should have a good idea of how to include the allowed foods in a satisfying and delicious way! You will then start to make your own meals getting some inspiration from your favorites.

**When you are cooking and assembling your own meals - how many grams of carbohydrate are recommended?**

The goal during this diet is to have your body switch to ketones as a source of fuel. Each one of you might achieve that with slightly different amounts of carbohydrates. You will be monitoring ketone levels (using a glucose/ketone meter) and keeping track of your dietary intake using a food logging app (Cronometer) which will help you tally the carbohydrates consumed. We will review these numbers with you and will help guide you to the right amount of carbohydrates for you.

**What do I need to know about the effect of the diet on my blood sugars?**

Carbohydrates are the nutrient that have the most impact on blood sugars. As you radically limit the intake of carbohydrates during the ketogenic diet, your blood sugars will decrease. If you are not taking medications for diabetes, your blood sugars will not drop to a dangerous level, but if you are taking diabetes medications, please be extra aware of any low blood sugar symptoms you might experience. These include: feeling shaky, nervous or anxious, sweating and/or chills, irritability, confusion, fast heartbeat, feeling lightheaded or dizzy. If at any point you have any of these symptoms, please check your blood sugar using the glucose meter provided. If blood sugar is below 70 mg/dl, please treat this by taking **one** of the following:

4 glucose tablets

½ cup of fruit juice

½ cup of regular soda

3-4 teaspoons of sugar or sugar packets (not Splenda or Stevia or any other sweetener)

Wait 15 minutes and check blood sugar again. If it is still low, treat again as above. If symptoms don’t stop, call your healthcare provider.

**Are there any side effects?**

For some people the switching of fuels will cause some side effects, especially during the first week. When you start to limit carbohydrates in the diet, you will be using the stored carbohydrates in your body. These are stored as glycogen. Glycogen is stored with a lot of water; for every gram of glycogen, you store 3-4 gr of water with it. As these glycogen stores are consumed, all that stored water is liberated and excreted.

As you limit carbohydrates in the diet, the circulating insulin levels will decrease. With lower insulin levels, the kidneys excrete more sodium in the urine, and the loss of sodium can in turn cause an imbalance of other minerals such as magnesium and potassium.

Some of the symptoms of switching to a ketogenic diet (explained below) are due to this loss of water and sodium.

- Symptoms might include: fatigue, headache, cramping, irritability, difficulty focusing, and heart palpitations. This is often refer to as the “keto flu”. Please do let us know if you are suffering from these symptoms. Some of these can be prevented or alleviated by ensuring that you are drinking enough water and including more salt. Try to include a minimum of 12 cups of water per day (about 100 oz). One way to add both water and salt is to drink a cup of bouillon, chicken or beef broth twice daily in addition to the other fluids you include in your day.
- Heart palpitations- **very important**: if you are taking a medication for diabetes or for blood pressure, please report this symptom to Dalia. The palpitation might be due to the decreased fluid in the blood and the heart having to compensate by beating stronger, and this symptom can be alleviated by drinking enough fluids accompanied by some salt. But for those taking medications, it can be a sign of low blood sugars (hypoglycemia) or blood pressure that is too low and a change in the medication dose is needed.
- Another symptom you might experience is constipation. As you eliminate whole grains, legumes, and fruits, you will be limiting sources of fiber in your diet. Drinking the amount of fluid described above will help. Additionally, try increasing the intake of vegetables. If needed, you can include psyllium husks as a source of fiber. You can add a tablespoon in a glass of water and drink that.
- Leg cramps- these are due to the mineral imbalance and depending on the severity, we can recommend specific foods to increase mineral intake. Additionally an electrolyte supplement might be recommended.
- Bad breath- As you accumulate ketones in your blood, some of these will also “spill” into the breath or sweat. Ketones smell “fruity” or like nail polish remover. This will probably resolve on its own.

**What about physical activity?**

During the first week on the keto diet, you might have to limit the amount of physical activity you include as your body is transitioning fuels and might not be ready to provide enough for a strenuous activity. You will probably have to slowly build your exercise tolerance while your body adapts to using ketones for fuel. Once it is adapted, you should be able to exercise for even longer periods than when you depended on glucose for fuel!

Fatigue might also be caused by not consuming enough calories. If you are limiting fat intake, consider increasing the use of avocados, cream, oils, and butter.

**Resources**

Dietdoctor.com

CGM and glucose meter instructions

Here is the manual: <https://s3-us-west-2.amazonaws.com/dexcompdf/G6-CGM-Users-Guide.pdf>

Video tutorials: <https://www.dexcom.com/guides>

**Questions or concerns?**

Alex Schiavuzzi - Study Coordinator – [aschiavu@standford.edu](mailto:aschiavu@standford.edu) - 650.725.9451
