# Supplementary material for: Adherence to Ketogenic and Mediterranean Study Diets in a Crossover Trial: The Keto–Med Randomized Trial
Source: Nutrients. 2021 Mar 17;13(3):967. doi: 10.3390/nu13030967 (PMC8002540; doi:10.3390/nu13030967)
Supplement: Supplementary file 1 [file nutrients-13-00967-s001.zip › Supplemental File 3.docx]

**Mediterranean Plus Diet**

**What is a Mediterranean diet?**

The Mediterranean diet is not a single prescribed diet, but rather a general food-based eating pattern, which is marked by local and cultural differences throughout the Mediterranean region. This type of eating pattern emphasizes plant food, fish and seafood, and uses olive oil as the main fat.

**What is the “Plus” added to the Mediterranean diet?**

For this study, we are making the already healthy Mediterranean diet even healthier by restricting added sugars and refined grains.

**What foods are included in the Mediterranean Plus diet?**

- Vegetables- all vegetables are encouraged!
- Fruits- in their whole form, no juices
- Legumes: lentils and beans of all types
- Seeds and nuts
- Whole grains: brown, red, or black rice, oats, quinoa, buckwheat, farro, only whole grain breads and pastas
- Main animal food: fish and seafood. Including these at least twice a week is recommended.
- Main fat: olive oil
- **
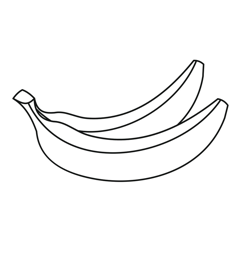

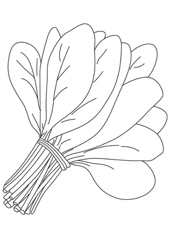
*
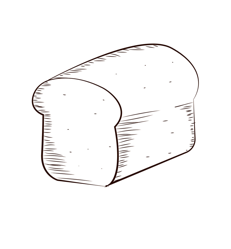
*
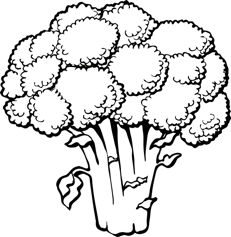
**Herb and spices: basil, dill, oregano, thyme, sage, mint, parsley, cilantro, garlic, paprika, nutmeg, cinnamon, etc.

***
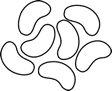
*
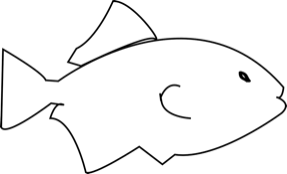
**

**What foods are limited in the Mediterranean Plus diet?**

- Dairy foods: choose mostly yogurt and cheese.
- Eggs
- Poultry
- Meat: beef, pork, lamb are only included in small portions and not very often

*
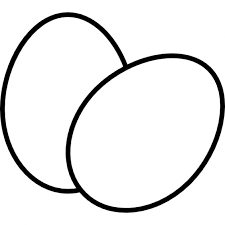
***
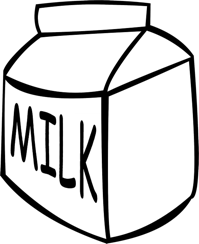
***
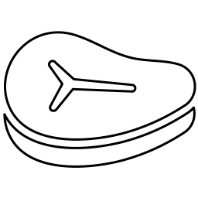
***
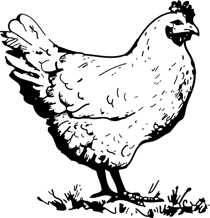
**

**What foods are not included in the Mediterranean Plus diet?**

- Sodas and juices. Also eliminate sports drinks, “Vitamin water” and other beverages that contain sugar
- Chocolate and other candies
- Pastries of all kind
- Refined grains: white bread, pasta made with refined wheat, white rice, etc.
- Processed meats: sausages, hot dogs, bacon, etc.

**
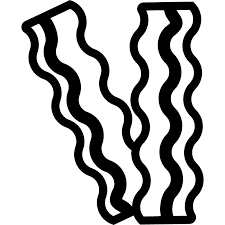
*
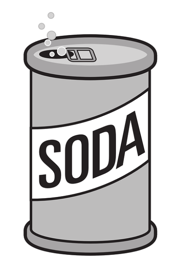

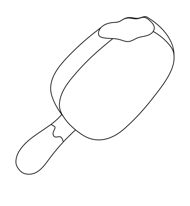
***

**
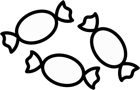

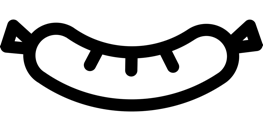
**

Here is a general guide for the proportions of food that should be in your diet. It is important to remember that although some foods are technically allowed on the Mediterranean Diet, they are *not* encouraged to be eaten frequently.


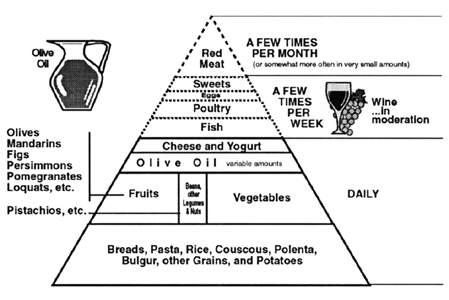


**Determining Whole Grains**

A grain is considered to be a “whole grain” when it has all three original parts: the bran, the germ, and the endosperm. If all three of those parts are present and in the same proportions as is found in nature as the grain is developing, then it is categorized as a whole grain. “Refined Grains” are grains that do not have all three of these parts. For example, white rice and white flour have both the bran and the germ removed, leaving only the endosperm. The removal of parts of the grain leads to the removal of anywhere between half to two-thirds of the nutrients. For the Mediterranean diet, it is important to only be eating Whole Grains so that you are getting all of the possible nutrients!

**What about alcohol?**

If you drink alcohol, we recommend that you drink in moderation and choose mostly red wine. If you don’t drink, don’t start!

**How do I follow this diet?**

The first 4 weeks, you will not have make many decisions, as you will be receiving your meals! During that period we will have opportunities to talk about shopping, cooking, eating out, etc. and you will feel ready to embrace this diet. We will have a combination of personal and virtual meetings to accommodate your needs and your schedule.

**What is included in my Methodology delivered meals?**

We worked very closely with the team at Methodology to develop the recipes for the foods you will receive. These meals follow the guidelines for a healthy, balanced Mediterranean diet. Each component of a meal is delivered in a glass jar and you can mix and match the content to make the meal of your choice. Some examples of what you will get may include Cinnamon Quinoa Porridge, Bruleed Pinneaple Overnight Oats, Pan-Seared Salmon, Jidori Za'atar Chicken Breast, Ginger Scallion Wild Black Cod, Mashed Cauliflower, Roasted Asparagus, salads, and more.

After the 4 weeks of delivered food, you should have a good idea of how to include the allowed foods in a satisfying and delicious way! You will then start to make your own meals getting some inspiration from your favorites.

**How do I maintain the carbohydrate/fat/protein balance once I am cooking on my own?**

You should try to limit the carbohydrate intake to 150 gr per day. By doing so, you will naturally consume more vegetables and more fat. Protein will most likely be maintained at the 15% of your calories without effort on your end.

When choosing fats, make sure you are including mostly olive oil (don’t be too shy when using this!), avocado, nuts, and seeds.

The tables below will help you tally the carbohydrates in your diet. Make sure your sources of carbohydrate are whole grains, legumes, fruits, starchy vegetables, nuts and seeds. No refined starches, and no sugars. You can also log your food in Cronometer to help you tally the carbohydrates up to 150 gr per day.

**VEGETABLES**

| **Low Carbohydrates**  **As much as you want** | **Medium Carbohydrates**  **Almost as much as you want**  **½ cup (cooked) have 5 gr** | **High Carbohydrates**  **Good choices to spend your carbohydrate budget.**  **½ cup (cooked) have 15 gr** |
| --- | --- | --- |
| Alfalfa seeds, sprouted | Beets | Blackeye peas |
| Artichoke globe, 1 unit | Brussels sprouts, 3 each | Beans: black, garbanzo, navy, pinto, red, and most other beans, cooked and drained |
| Arugula | Carrots | Corn |
| Asparagus | Leeks | Edamame |
| Beet greens | Mushrooms | Jerusalem artichokes |
| Broccoli | Onions | Kohlrabi |
| Cabbage | Pepper, red | Lentils |
| Cauliflower | Shallots | Lima beans |
| Celery | Jicama | Lotus root |
| Chard |  | Parsnip |
| Collard greens |  | Peas |
| Cilantro, free |  | Pickles, sweet |
| Cucumber |  | Potato |
| Dandelion greens |  | Pumpkin |
| Eggplant |  | Rutabaga |
| Fennel |  | Soybeans |
| Ginger root, free |  | Squash, winter (acorn, butternut, spaghetti) |
| Green beans |  | Sweet potato |
| Hearts of Palm |  | Tomato, juice or canned |
| Kale |  | Vegetable juice |
| Lettuce |  | Water chestnut |
| Mung beans, sprouted |  | Yam |
| Mustard greens |  |  |
| Nopales |  |  |
| Okra |  |  |
| Parsley, free |  |  |
| Pepper, green |  |  |
| Pickles, dill |  |  |
| Radicchio, free |  |  |
| Radish |  |  |
| Sauerkraut |  |  |
| Seaweed, dried |  |  |
| Snap peas |  |  |
| Spinach |  |  |
| Tomato, fresh |  |  |
| Turnip |  |  |
| Turnip greens |  |  |
| Watercress, free |  |  |

**FRUIT**

|  | **Amount** | **Carbohydrate grams** |
| --- | --- | --- |
| Apple | 4 oz | 15 |
| Applesauce | ½ cup | 15 |
| Apricots, dried | 7 pieces | 15 |
| Banana | 6” | 15 |
| Blackberries | ½ cup | 10 |
| Blueberries | ½ cup | 10 |
| Cantaloupe, Honeydew | 1 cup | 15 |
| Cherries | 12 | 15 |
| Date, Medjool | 1 | 15 |
| Grapefruit | ½ large | 15 |
| Grapes | 15 small | 15 |
| Kiwi | 1 | 15 |
| Orange | 1 medium | 15 |
| Pear | 6 oz | 15 |
| Pineapple | 1 cup | 20 |
| Prunes, dried | 3 | 15 |
| Raisins | 1/8 cup | 15 |
| Raspberries | 1 cup | 15 |
| Strawberries | 1 cup | 12 |
| Watermelon | 1 cup | 12 |

**DAIRY**

|  | **Amount** | **Carbohydrate grams** |
| --- | --- | --- |
| Cottage cheese | ½ cup | 2-6 |
| Ricotta cheese | ½ cup | 3-6 |
| Milk (whole, low fat, or non-fat) | 1 cup | 12 |
| Yogurt- plain or “light” | 8 oz | 12-16 |
| Greek style yogurt- plain | 8 oz | 6-7 |
| Greek style yogurt- flavored | 6 oz | 12-20 |
| Buttermilk | 1 cup | 12 |

**GRAINS**

|  | **Amount** | **Carbohydrate grams** |
| --- | --- | --- |
| Most cooked grains (oatmeal, quinoa, farro, barley), except rice | ½ cup | 15 |
| Rice | ⅓ cup | 15 |
| Breads | 1 oz (1 slice) | 15 |

**What do I need to know about the effect of the diet on my blood sugars?**

Carbohydrates are the nutrient that have the most impact on blood sugars. As you limit the intake of sugars and refined flours during the Mediterranean Plus diet, your total intake of carbohydrates will be lower. If you are not taking medications for diabetes, your blood sugars will not drop to a dangerous level, but if you are taking diabetes medications, please be extra aware of any low blood sugar symptoms you might experience. These include: feeling shaky, nervous or anxious, sweating and/or chills, irritability, confusion, fast heartbeat, feeling lightheaded or dizzy. If at any point you have any of these symptoms, please check your blood sugar using the glucose meter provided. If blood sugar is below 70 mg/dl, please treat this by taking **one** of the following:

- 4 glucose tablets
- ½ cup of fruit juice
- ½ cup of regular soda
- 3-4 teaspoons of sugar or sugar packets (not Splenda or Stevia or any other sweetener)

Wait 15 minutes and check blood sugar again. If it is still low, treat again as above. If symptoms don’t stop, call your healthcare provider.

**What about physical activity?**

You are encouraged to include physical activity during the study. Be aware that being physically active may lower blood sugars. Please refer to the section above if you have any of the symptoms of low blood sugar when you exercise.

**Resources**

<https://oldwayspt.org/traditional-diets/mediterranean-diet>

<https://www.healthline.com/nutrition/mediterranean-diet-meal-plan>
