# Supplementary material for: Adherence to Ketogenic and Mediterranean Study Diets in a Crossover Trial: The Keto–Med Randomized Trial
Source: Nutrients. 2021 Mar 17;13(3):967. doi: 10.3390/nu13030967 (PMC8002540; doi:10.3390/nu13030967)
Supplement: Supplementary file 1 [file nutrients-13-00967-s001.zip › Supplemental File 4.docx]

**Keto Handout**

If you are receiving this handout, you are ready to start preparing *your own* food. The Methodology food was delicious, but now it’s time for you to utilize your own culinary skills. This handout will include:

- General overview of Do’s and Don’ts
- A custom shopping list filled with delicious food options
- Meal ideas
- How to get your pantry ready for the next 8 weeks.

**General Concepts**

Do not be afraid of fats. You will be using these as a source of fuel and will need these to have energy.

Be moderate in protein. Excess protein can be transformed into glucose (sugar) in the body.

Keep carbohydrates as low as you can.

Choice order of oils: olive, high-oleic safflower, canola are the preferred oils. Other preferred sources of fat: avocado, olives, and nuts (in moderation as these provide some carbs and lots of protein).

Then dairy and meat fats.

Last: soybean, corn, sunflower, cottonseed oil.

**
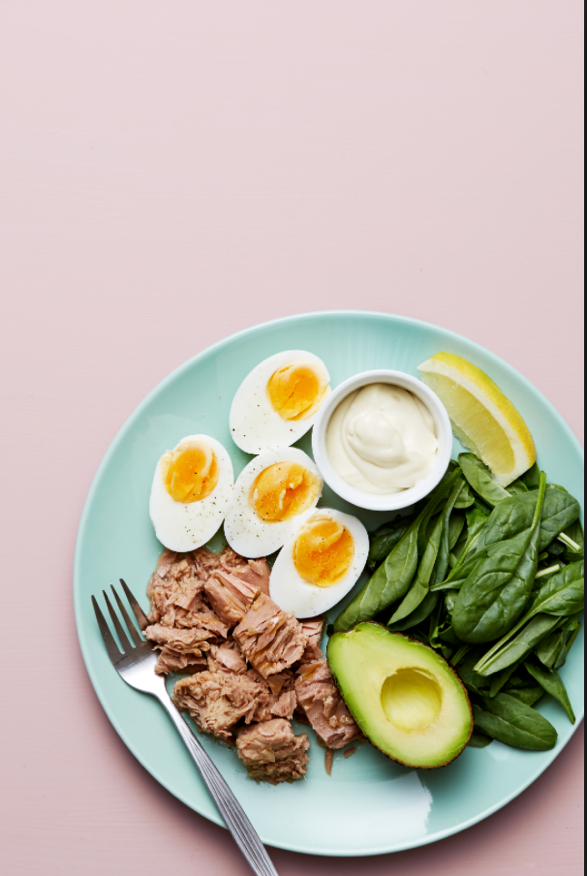
**Make sure you include salt, especially on hot days and before exercise. If you are feeling fatigued, you can include Bouillon cubes – 2 per day or make your own broth with I tsp salt per quart. This helps prevent dehydration.

**Get Your Pantry/Fridge Ready**

Meat

- Chicken thighs
- Pork (yes this includes bacon)
- Steak
- Salami
- Any cut of meat, preferably something higher in fat

Fish (canned options good for making tuna or salmon salad)

- Tuna
- Salmon
- Mackerel
- Haddock

Dairy

- Cheeses (opt for high fat cheeses like cream cheese)
- Heavy Cream (no ½ and ½)
- Butter
- Ghee

Other

- Eggs
- Olive oil
- Seasonings
- VEGGIES! Choose veggies that are grown above ground. Exclude starchy root vegetables like potatoes, beets and yams.
- Very Very dark chocolate in small amounts (>90% cocoa)
- Berries in small amounts

**Keto Diet Meal Ideas**

(some are from “The Art and Science of Low Carbohydrate Living” by Volek and Phinney)

Breakfast:

- Eggs with veggies, some bacon, some cheese
- Smoked salmon, cream cheese, lettuce wrappers, capers
- Ham wrapped asparagus
- Cauliflower beef hash with peppers and onions

Lunch:

- Tuna, mixed greens, olives, blue cheese dressing
- Beef broth, roast pork, lettuce wedge, honey basil dressing
- Chicken Caesar salad (no croutons)
- Cobb salad (no corn)
- Sautéed spinach, green beans, avocado, and roasted chicken thighs
- No bun burger, salad

Dinner:

- Steak, broccoli with butter, mushrooms, tomato bisque with cream
- Chicken with fennel, kale, sautéed with bacon, garlic and olive oil
- Back pork ribs, lettuce wedge, creamy dressing, tomatoes and cucumber
- French onion soup (no bread), fish with creamed spinach
- Sausage chili, asparagus with herb butter
- Roasted chicken and salad

Snacks:

- Broth
- Celery with cream cheese
- Cucumber with high fat yogurt dressing
- Olives
- Mozzarella cheese balls with cherry tomatoes, basil, and olive oil and balsamic vinegar
- Hardboiled egg or egg salad
- Tuna with cream cheese
- Kale chips
- Keto sushi roll: Nori sheet with avocado or cream cheese, optional fish, raw veggies- rolled tightly and cut
- Veggie sticks with guacamole
- Fermented and or pickled veggies
- “Fat bombs”

**Resources for More Recipes**

- LCHF_Italian YouTube channel has easy and delicious meal ideas and recipes.
  - <https://www.youtube.com/channel/UCmJTEfBXR9a4LQ5PeaN9qJg>
- Epicuriuos site has a Ketogenic list of great recipes
  - <https://www.epicurious.com/expert-advice/keto-diet-recipes-that-are-actually-delicious-gallery>
- Diet Dr. has a large variety of recipes that are delicious
  - <https://www.dietdoctor.com/low-carb/recipes>
