# Supplementary material for: Adherence to Ketogenic and Mediterranean Study Diets in a Crossover Trial: The Keto–Med Randomized Trial
Source: Nutrients. 2021 Mar 17;13(3):967. doi: 10.3390/nu13030967 (PMC8002540; doi:10.3390/nu13030967)
Supplement: Supplementary file 1 [file nutrients-13-00967-s001.zip › Supplemental File 5.docx]

**Mediterranean Handout**

If you are receiving this handout, you are ready to start preparing *your own* food. The Methodology food was convenient, but now it is time for you to utilize your own culinary skills. This handout will include:

- Target portions and servings
- How to get your pantry ready for the next 8 weeks.
- A custom shopping list filled with delicious food options
- Meal ideas

Servings per Day

- Grains, starchy veggies (potato, yam, corn): 2-3 servings per day
- Beans and peas: 1-3 servings per day
- Fruits: 2-3 servings per day
- Vegetables: > 4 servings per
- Fish: 2-3 servings per week
- Poultry: 0-2 times per week
- Red meat: <1 per week
- Eggs: 0-4 eggs per week
- Dairy:
  - Yogurt: 3-5 per week
  - Cheese: 0-5 per week

What does **one** serving look like?

- Grains: ½ cup cooked, 1 slice of whole wheat bread, ½ cup of whole wheat pasta; potato 1 cup cooked and corn ½ cup
- Beans and peas: ½ cup cooked
- Fruits: 1 cup
- Vegetables: 1 cup raw, ½ cup cooked
- Fish: 4 oz.
- Poultry: 4 oz.
- Red meat: 4 oz.
- Eggs: 1 egg
- Dairy
  - Yogurt: 6-8 oz.
  - Cheese: 1 oz

Discouraged from diet:

- All sugars and refined starches: soda, sweetened beverages, white bread, white pasta, white rice, pastries, other sweet snacks or desserts.
- Processed meats: bacon, deli meats

**Get Your Pantry/Fridge Ready**

Meat

- Chicken
- Turkey

Oily fish (canned options good for making tuna or salmon salad)

- Tuna
- Salmon
- Mackerel
- Haddock

Shell fish

- Shrimp
- Prawns

Bread and Grains (TIP: look for labels that say 100% Whole Grain)

- Whole grain bread
- Whole grain pasta
- Farro
- Quinoa
- Roasted buckwheat
- Wild rice
- Brown rice

Legumes

- Lentils
- Chickpeas
- Pinto beans
- Black beans
- Any other bean you like. Canned or dried for cooking are all good

Nuts and seeds

- Any that you like- almonds, pecans, walnuts, sesame seeds, chia seeds, sunflower seeds, pumpkin seeds (pepitas), hummus, roasted chickpeas

Dairy

- Low fat milk (If you use milk)
- Unsweetened yogurt
- Feta cheese
- Mozzarella cheese

Other

- Eggs
- Olive oil
- Balsamic vinegar
- Veggies and fruits: whatever is fresh, seasonal and looks and smells good. **Get lots of produce!**

**Mediterranean Diet Meal Ideas**

All meals with a * have a recipe provided on the following pages

Breakfast:

- Overnight oats *
- Steal cut oats with fruit
- Omelet with veggies
- Hardboiled egg with a chopped salad of tomatoes, cucumbers, scallions, radishes with olive oil and lemon juice

Lunch

- Lentil salad*
- Cobb Salad*
- Whole wheat wrap filled with roasted veggies and hummus
- Vegetable soup and a big salad

Dinner- make the veggies center stage!

- Roasted chicken, big salad, roasted potatoes
- Roasted mixed veggies (Broccoli, cauliflower, onion, sweet potato, carrots, whatever you want), cooked Farro (or any other whole grain), pan fried shrimp
- Lentil soup* and a big salad
- Roasted salmon with asparagus and a side of polenta

Snacks

- Veggies and Hummus
- Veggies and Guacamole
- Fruit
- Caprese salad*
- Quinoa salad (similar to lentil salad🡪 replace lentil with quinoa)
- Oven roasted chickpeas*
- Kale chips*

***Recipes**

**Overnight oats**

Ingredients

- 1-part Oats
- 1-part Almond milk
- ¼ - part Chia seeds
- ½ of shredded or diced apple (or other choice of fruit)
- Dash of Cinnamon

Directions

1. Put oats, almond milk, and chia seeds in a bowl.
2. Mix together
3. Place in fridge overnight
4. Add apples, cinnamon, or any other toppings when ready to eat

**Lentil Salad**

Ingredients:

- 1 Cup Dried Lentils
- ¼ cup Red Onions (diced)
- ¼ cup Parsley (diced)
- ¼ cup Kalamata olives (chopped)
- 1 cup Cucumber (chopped)
- 1 Cup Tomatoes (chopped)
- 1/3 cup Feta cheese (optional)
- Olive oil/vinaigrette 🡪 If you buy dressing, make sure to check the ingredients. A lot of dressings contain sugars.

Directions:

1. Prepare dried lentils according to package
2. Cut up and prepare all vegetables
3. Toss all ingredients in a large mixing bowl

**Salad Dressing**

Ingredients:

- 2 Tbsp Olive Oil
- 1 Tbsp Balsamic Vinegar
- 2 Tsp Dijon Mustard
- 1-2 Tbsp Minced scallions
- Touch of honey
- Dash Salt/pepper

Directions:

1. Mix all ingredients together in a cup or bowl

**Cobb Salad**

Ingredients:

- ½ Cup roasted chicken, shredded
- 1-2 Hard-boiled eggs
- Large Bowl filled with mixed greens
- 1 heirloom tomato cut up
- ¼ cup cucumbers cut
- ½ Avocado
- ¼ cup red onions chopped
- Any other veggie of your choice
- Blue cheese (optional)
- Vinaigrette

Directions:

1. Wash and cut all veggies
2. Toss in a large bowl with dressing and enjoy!

**Caprese Salad**

Ingredients:

- 1-2 Heirloom Tomatoes
- Fresh Mozzarella (buffalo Mozzarella from Costco is delicious)
- Handful of fresh basil
- Olive oil/ balsamic vinegar

Directions:

1. Slice heirloom tomatoes
2. Slice mozzarella
3. Lay tomatoes and mozzarella slightly overlapping on the plate, alternating tomato and cheese
4. Sprinkle basil on top
5. Drizzle olive oil and balsamic vinegar

**Oven roasted Chickpeas**

Ingredients:

- 1 can Chickpeas
- 2 tbsp Olive Oil
- Dash of Salt
- Any spices you want (suggestions below)
  - Chili powder and lime
  - Garlic powder
  - Zaatar
  - Rosemary
  - Any other spice or herb that you like

Directions:

1. Preheat oven to 450 degrees F
2. Drain chickpeas from can
3. Blot with paper towel to partially dry them, and place in bowl
4. Toss with olive oil and seasoning of choice.
5. Spread on baking sheet and bake for 30-40 mins until chickpeas are golden and crunchy.

**Kale chips**

Ingredients:

- One bunch of kale
- 2 tbsp olive oil
- Dash of salt
- Any spices you want

Directions:

1. Preheat oven to 300 degrees F
2. Toss kale in olive oil, salt, and any spices
3. Lay flat on baking pan.
4. Bake for about 20 mins until kale is light and crunchy.

**Red Lentil Soup (from: Bakerita.com)**

Ingredients:

2 tablespoons avocado oil or olive oil

1 small onion diced

1 large carrot diced

2 stalks of celery diced

4 cloves garlic crushed

1 teaspoon freshly grated ginger

1 teaspoon cumin

1 teaspoon turmeric

1 teaspoon garlic powder

1 teaspoon ginger powder

1 teaspoon salt

4 cups vegetable broth

2 cups water

1 (15 oz.) can crushed tomatoes

1 1/2 cups red lentils sort and rinse before using

3 cups spinach or mix of greens I used a “power greens” blend of spinach, arugula and baby chard

1 lemon zest and juice

To garnish

Lemon wedges

Microgreens

Instructions:

In a soup pot, heat the avocado or olive oil over medium heat. Once the oil is shimmering, add the onion, carrot, and celery and cook for about 5 minutes, until the onion is translucent.

Add the garlic, grated ginger, cumin, turmeric, garlic powder, ginger powder, and salt, and stir to combine and coat all the veggies in the spices.

Let cook for 2 minutes, until fragrant, and then add the vegetable broth, water, tomatoes, and red lentils. Stir and bring to a boil, then turn down and let simmer for 30-40 minutes, until the red lentils are soft. Taste, and add more salt to your tastes if desired.

If desired, puree half the soup to help make it creamier. You can also just leave it as is if you prefer a more broth-y soup. I pureed some of mine with an immersion blender.

Stir in your greens and let them wilt, and then add in lemon juice and zest. Serve with more lemon if desired (I recommend), and garnish with some microgreens!

**Resources for More Recipes**

The following websites are there to give you ideas for meals that include a lot of vegetables and well spiced for flavor. For some of these you should substitute the grains for whole grains (i.e. if the recipe calls for white rice, use brown or if it calls for regular pasta, choose whole wheat pasta).

Choose those recipes with fish and sometimes chicken, and leave the beef or lamb for no more than once per week.

Also, stay away from desserts with added sugars. You can have some unsweetened yogurt with fresh or cooked fruit, baked apple or pear, or a small piece of very dark chocolate.

Get inspired and make some delicious meals!

- <https://cookieandkate.com/> delicious vegetarian recipes to make your meals with a large percentage of veggies and beans
- <https://oldwayspt.org/recipes> a site with lots of information about the Mediterranean diet. Also has good recipes
- https://www.themediterraneandish.com/
- An idea for meal prepping: <https://ohsheglows.com/2019/03/07/meal-prep-week-long-power-bowls/>

An interesting write-up about the Mediterranean Diet

<https://www.truehealthinitiative.org/news2019/is-the-mediterranean-diet-the-best-choice-for-health/>
