# Supplementary material for: Adherence to Ketogenic and Mediterranean Study Diets in a Crossover Trial: The Keto–Med Randomized Trial
Source: Nutrients. 2021 Mar 17;13(3):967. doi: 10.3390/nu13030967 (PMC8002540; doi:10.3390/nu13030967)
Supplement: Supplementary file 1 [file nutrients-13-00967-s001.zip › Supplemental File 6.docx]

Food Satisfaction

The Food Satisfaction Questionnaire was administered at 2 timepoints during the study–at the end of each phase (Visits 4 & 6). The 2^nd^ timepoint contained a few additional questions comparing the two diets.

Purpose: To assess participants’ level of satisfaction of each diet, both while on Methodology food and when providing their own food. Questions on feasibility and preparedness to follow the diets were also included. The questionnaire at the end of Phase 2 asked the participants to compare the 2 diets in terms of taste, feasibility and cost.

Scoring: Questions relating to diet satisfaction can be scored using a 1-5 scale, with a higher number indicating greater satisfaction. Questions relating to comparing the 2 diets can be scored using a 1-5 scale, with an average value of 3 indicating equal satisfaction with the diets, <3 indicating preference for the Ketogenic Diet and >3 indicating preference for the Mediterranean Diet.

METHODOLOGY PHASE (first 4 weeks)

These questions refer to the first 4 weeks of this phase of the study where you received ALL your food from Methodology.

1. For this phase, what diet were you on?
   1. Ketogenic
   2. Mediterranean
2. How would you rate the overall level of service (e.g., delivery, packaging, labeling) from Methodology?
   1. Not at all satisfied
   2. Slightly satisfied
   3. Moderately satisfied
   4. Very satisfied
   5. Extremely satisfied
3. How would you rate the overall taste of Methodology food products?
   1. Poor
   2. Fair
   3. Good
   4. Very good
   5. Excellent
4. How would you rate the overall quality (e.g., freshness, appearance) of Methodology food products?
   1. Poor
   2. Fair
   3. Good
   4. Very good
   5. Excellent

PROVIDING YOUR OWN FOOD (last 2 months)

These questions refer to the two months of this phase when you were not longer receiving any food

1. During this phase, how well do you feel you were prepared for and understood what food and beverages to include and avoid?
   1. Very unprepared
   2. Somewhat unprepared
   3. Intermediate
   4. Adequately prepared
   5. Very well prepared
2. Prior to starting the study, how often did you or someone in your family cook and prepare your meals for you, relative to eating out, or ordering take-out?
   1. Almost always got meals from outside my home and/or take-out
   2. Frequently got meals from outside my home and/or take-out
   3. Half & half
   4. Frequently ate meals prepared at home
   5. Almost always ate meals prepared at home
3. During the study, how often did you or someone in your family cook and prepare your meals for you, relative to eating out, or ordering take-out?
   1. Almost always got meals from outside my home and/or take-out
   2. Frequently got meals from outside my home and/or take-out
   3. Half & half
   4. Frequently ate meals prepared at home
   5. Almost always ate meals prepared at home
4. For any meals you ate that were prepared at home, who was primarily responsible for those meals?
   1. Someone else at home made almost all my meals for me
   2. Someone else at home made most of my meals for me
   3. Half & half
   4. I made most of the meals I ate that were prepared at home
   5. I made almost all of the meals I ate that were prepared at home
5. How hard vs. easy did you find it to adhere to the study diet guidelines on your own (after having everything made for you by Methodology and delivered)?
   1. Very hard
   2. Hard
   3. Intermediate
   4. Easy
   5. Very easy
6. How much did you enjoy the taste and variety of the foods for your study diet that you were responsible for on your own (i.e., after Methodology)?
   1. Disliked
   2. Somewhat disliked
   3. OK
   4. Liked
   5. Liked very much
7. Compared to how much you spent of food before the study started, what was the cost of the study foods like for you when you were responsible for getting them on your own (i.e., after Methodology)?
   1. Much more expensive
   2. More expensive
   3. Similar
   4. Less expensive
   5. Much less expensive

ADDITIONAL QUESTIONS (ASKED ONLY AFTER THE SECOND DIET PHASE)

For the final questions, we will ask you to compare the two phases of the study.

1. Reflecting on the first 4 weeks of each phase of the study (i.e.: while receiving food from Methodology), in terms of TASTE and ENJOYMENT:
   1. I greatly preferred Keto to Med
   2. I somewhat preferred Keto to Med
   3. I found the two equal in terms of taste and enjoyment
   4. I somewhat preferred Med to Keto
   5. I greatly preferred Med to Keto
2. Reflecting on the last 2 months of each phase of the study, when I was responsible for my own food, in terms of TASTE and ENJOYMENT:
   1. I greatly preferred Keto to Med
   2. I somewhat preferred Keto to Med
   3. I found the two equal in terms of taste and enjoyment
   4. I somewhat preferred Med to Keto
   5. I greatly preferred Med to Keto
3. Reflecting on the last 2 months of each phase of the study, when I was responsible for my own food, in terms of HOW EASY IT WAS TO FOLLOW MY DIET SOCIALLY (family, friends, work, etc):
   1. Keto was much easier than Med
   2. Keto was somewhat easier than Med
   3. I found the two equal
   4. Med was somewhat easier than Keto
   5. Med was much easier than Keto
4. Reflecting on the last 2 months of each phase of the study, when I was responsible for my own food, in terms of HOW EXPENSIVE IT WAS TO FOLLOW MY DIET:
   1. Keto was much less expensive than Med
   2. Keto was somewhat less expensive than Med
   3. I found the two equal
   4. Med was somewhat less expensive than Keto
   5. Med was much less expensive than Keto

Diet Satisfaction

The Dietary Satisfaction Questionnaire (DSat-28©) was administered at 4 time points during the study:

1.Visit 2 -Baseline

2.Visit 4 -End of diet 1

3.Visit 6 -End of diet 2

4.Visit 7 -Washout

Purpose: To determine to what extent participants were satisfied with various aspects of their diets before being in the study, during each phase, and during their chosen diet in washout

Scoring: Scales are measured using five responses ranging from 'Disagree Strongly' to ‘Agree Strongly’ which are scored from 1 to 5. Items are reverse scored if necessary, so that higher scores indicate greater diet satisfaction. Item scores are averaged to provide scale scores.

The full questionnaire is available at the reference below.

Reference: Validation of the Diet Satisfaction Questionnaire: a new measure of satisfaction with diets for weight management. *Obes Sci Pract*. 2018 Oct 10;4(6):506-514.

<https://pubmed.ncbi.nlm.nih.gov/30574344/>

COVID Related Alterations

The COVID Questionnaire was administered to participants after they attended their final washout blood draw.

Purpose: The purpose of the questionnaire was to find out if a participant ever tested positive for, or had any reason to believe, that they had COVID-19 at any point while in the Keto-Med study. This was crucial information to have for individuals handling participant blood and stool samples upon study completion.

---------------------------------

Dear participant,

We are well aware and appreciative of the challenges of being in a research study during these past several months. As we have learned from speaking with many of you, some have had their lives disrupted more than others. When we publish the results of the study, it will be important for us to document how this once-in-a-lifetime global situation impacted participants in the study.

Please provide responses to the following to help us capture this information:

1. Please rate your ability to follow your assigned diet during the shelter in place (relative to how well you were able to follow it prior to the shelter-in-place orders).
   100% = same adherence, no impact of the shelter in place
   0% = not following the study protocol at all during the shelter in place:
   1. 100%
   2. 90%
   3. 80%
   4. 70%
   5. 60%
   6. 50%
   7. 40%
   8. 30%
   9. 20%
   10. 10%
2. To what extent was your ability to follow the study diet impacted by changes in your access to food during the shelter-in-place?
   1. Greatly impacted
   2. Somewhat impacted
   3. Not at all impacted
3. To what extent did stressors (e.g., concerns, anxiety, fears, family or roommate challenges) impact your ability to follow the study protocol?
   1. Greatly impacted
   2. Somewhat impacted
   3. Not at all impacted
4. To what extent was your physical activity impacted by changes during the shelter-in-place?
   1. My physical activity increased
   2. My physical activity decreased
   3. My physical activity didn't change, but I changed activities
   4. No change
5. Is there any other information you would like to, or be willing to share about the challenges of following the study protocol during shelter-in-place?
